# A motivational approach to perfectionism and striving for excellence: Development of a new continuum-based scale for post-secondary students

**DOI:** 10.3389/fpsyg.2022.1022462

**Published:** 2022-11-10

**Authors:** Marie Lasalle, Ursula Hess

**Affiliations:** ^1^Department of Psychology, University of Quebec in Montreal, Montreal, QC, Canada; ^2^Department of Psychology, Humboldt University, Berlin, Germany

**Keywords:** perfectionism, self-determination theory, self-definition, exploratory structural equation modeling (ESEM), time invariance, scale construction

## Abstract

Perfectionism has been the object of many disputes. One such debate pertains to the nature of perfectionistic strivings. Whereas perfectionistic concerns (PC) have been shown to correlate with negative outcomes, perfectionistic strivings (PS) have been associated with mixed outcomes. This view of perfectionism assumes a motivational perspective; however, commonly used questionnaires assess motivation only implicitly. To create a more explicit measure of motivation as regard perfectionism, we aimed to assess perfectionism in post-secondary education based on Deci and Ryan’s self-determination continuum. We posit that introjected motivation represents the variance common to both dimensions of perfectionism. External motivation is considered to be specific to PC and identified motivation to PS. Amotivation represents a lack of meaningful striving. Intrinsic motivation, lacking perfectionism’s pressure, is conceptualized to be a self-determined form of striving for excellence. We further posited that this continuum is implicitly underlain by a continuum of self-definition as defined by Blatt. The resulting questionnaire showed an adequate structure with ESEM, followed a simplex structure, and had adequate reliabilities (Study 1a/Study 2). It also showed adequate convergent validity (Study 1b/Study 2). Finally, the questionnaire proved to be invariant over a 6-week period (Study 2). Results suggest that the degree of active goal pursuit, in addition to standards setting, could be a distinguishing characteristic between dimensions of perfectionism as well as striving for excellence.

## Introduction

Perfectionism is a trait people profess a need to have (Hill et al., 2015). It is currently understood as a complex multidimensional and interactive trait (Stoeber and Otto, 2006; Gaudreau and Thompson, 2010), characterized by a striving for perfection, extremely high standards for oneself, and stringent self-evaluations (Stoeber, 2018). Perfectionism has been studied specifically in the educational context with regard to both teachers (Stoeber and Rennert, 2008; Gluschkoff et al., 2017; Samfira and Paloş, 2021) and students (Stoeber et al., 2009; Milyavskaya et al., 2014; Madigan, 2019). Yet, perfectionism also has many negative consequences (Hill and Curran, 2015; Limburg et al., 2017; Sirois and Molnar, 2017; Smith et al., 2018). In the last 50 years, a wide range of theories–from early clinical theories to multiple research approaches that focus on definitions, correlates, and outcomes of the trait–have been developed to better understand this personality trait. Based on factor analyses of frequently used questionnaires, such as the Hewitt and Flett Multidimensional Perfectionism Scale (HF-MPS; Hewitt and Flett, 1991), the Frost Multidimensional Perfectionism Scale (F-MPS; Frost et al., 1990), and the Almost Perfect Scale (APS-R; Slaney et al., 2001), two factors have been suggested (Frost et al., 1993; Slaney et al., 1995; Dunkley et al., 2000; Cox et al., 2002; Bieling et al., 2004). A first dimension of perfectionism, studied under such names as socially prescribed perfectionism (SPP; Hewitt and Flett, 1991), perfectionistic concerns (PC; Stoeber and Otto, 2006), or evaluative concerns perfectionism (ECP; Dunkley et al., 2000) is characterized by the perceived imposition of high standards by the environment and a fear of others’ negative evaluations, as well as concerns over mistakes, doubts about actions, and perceived discrepancy between standards and performance. The subscales composing this dimension of perfectionism resemble early clinicians’ (Horney, 1950; Missildine, 1963; Hollender, 1965; Burns, 1980) descriptions of the trait, which include the pursuit of extremely high standards irrespective of situational demands, a lack of flexibility, and a lack of satisfaction derived from both the achievement process and its outcome. From this view, perfectionists are motivated by a desire to gain acceptance from others, or to protect the self from negative self-evaluations—and the associated emotions—that result from a conditional self-esteem.

The second dimension of perfectionism, studied under names, such as self-oriented perfectionism (SOP; Hewitt and Flett, 1991), perfectionistic strivings (PS; Stoeber and Otto, 2006), or personal standards perfectionism (PSP; Dunkley et al., 2000) is defined by the imposition of high and rigid standards on oneself, conjointly with stringent self-evaluations. Some (Slade and Owens, 1998; Stoeber and Otto, 2006) have described this dimension as a positive form of perfectionism, relating it to characterization of the trait of Hamachek (1978). Hamachek’s description of the trait features flexibility, consideration for one’s strengths and weaknesses in the striving process, and an outlook centered on seeking success instead of fearing failure. However, this description overlaps with other concepts such “master of his craft,” a healthy form of striving for excellence (Missildine, 1963, Chapter 10).

A first debate concerns therefore the difference between perfectionism and striving for excellence. Perfectionism has been hypothesized to be distinguishable from striving for excellence by the intensity of the standards espoused and of their pursuit, qualified by adjectives, such as excessive, exceeding, and relentless. As such, while perfectionists reach excellence, they cannot enjoy any sense of satisfaction, but instead must push further toward perfection (Gaudreau, 2019). However, it has also been suggested that it is not the exacting standards, but rather the conditional self-acceptance and negative self-evaluations which are at the core of perfectionism (Greenspon, 2000).

A second debate concerns the existence of a positive form of perfectionism. Some researchers consider perfectionism to be always detrimental (Hewitt et al., 2017; Smith, 2018). Conversely, other lines of research have suggested that controlling for the overlapping variance between dimensions (by partialling out overlapping variance or creating profiles) allows the emergence of a dimension of perfectionism that is more strongly associated with positive outcomes (Stoeber and Otto, 2006; Gaudreau and Thompson, 2010; Hill et al., 2010; Gotwals et al., 2012; Stoeber and Gaudreau, 2017). Yet, this position has been criticized on the grounds of theoretical and measurement concerns (Hill, 2014, 2017; Smith and Saklofske, 2017). Furthermore, even when these approaches are used, PS remains linked to negative outcomes and correlates in some settings (Saboonchi and Lundh, 2003; Molnar et al., 2012; Limburg et al., 2017), indicating that this dimension can also become detrimental to the individual under certain circumstances.

In this vein, much of the research on perfectionism tends to conceive of perfectionism less as an overarching generalized trait and more of a dynamic personality trait that is influenced by contextual specificities. As such, it has been suggested that the perfectionism disposition may vary through the lifetime, for example decreasing in intensity in older age (Chang, 2000; Landa and Bybee, 2007). Crucially, it has been shown to vary between life domains (Dunn et al., 2005; Stoeber and Stoeber, 2009; Franche and Gaudreau, 2016; Levine and Milyavskaya, 2018). After all, a person may wish to present impeccable work in order to compete with their peers for a spot in higher education, as well as because they would feel negatively about themselves otherwise, but not feel such pressure in the pursuit of a hobby. This is not without similarities with motivation research, which posits that humans pursue endeavors for reasons that vary widely and concomitantly (Deci and Ryan, 2000; Elliot and McGregor, 2001). For example, a successful professor would be high in achievement motives in their research, but also require social power motives when managing the students and assistants in their laboratory (McClelland, 1970).

In this vein, it has been suggested that dimensions of perfectionism may be differentiated by their core motivational forces (Hewitt and Flett, 1991). Likewise, perfectionism might be understood as a motive disposition, with PS representing autonomous forms of motivation, and PC controlled forms of motivation (Stoeber et al., 2018). More importantly, a closer inspection of previous perfectionism questionnaires reveals items that already refer to the motivation structure proposed by Deci and Ryan (1985b), but do not exactly fit the autonomous/PS and controlled/PC pattern. Hence, the creation of a measure of perfectionism that explicitly reflects the underlying motivational dimension seems indicated. This is the goal of the present research. Before describing the construction of the scale, we will briefly outline self-determination theory.

### A self-determination theory view of perfectionism

Self-determination theory (SDT; Deci and Ryan, 1985b, 2000; Ryan and Deci, 2000a,b) distinguishes between intrinsic motivation and several forms of extrinsic motivation. Intrinsic motivation refers to the pursuit of an activity for the challenge or the satisfaction it brings in itself, without bearing on the instrumental value of the pursuit. Extrinsic motivation is defined by the pursuit of an activity toward the attainment of a certain outcome. It is built on a continuum of behavior regulation, in which motivation becomes increasingly internalized, that is, closer to the individual’s values, needs, or goals. This continuum regroups four motivations: external, introjected, identified, and integrated motivation. External motivation is fueled by perceived external contingencies, leading to punishment avoidance or reward seeking. When these perceived external contingencies are internalized, without becoming part of the self, the individual is driven by introjected motivation. This form of behavior regulation is associated with self-conscious emotions, such as guilt, shame, or pride. It thus refers to self-evaluation and ego related goals. Further internalization of the behavior by the individual leads to identified motivation. The first step of this process is accomplished when the behavior becomes important for the individual. The behavior is thus increasingly self-regulated, but it still remains a means to an end. The last and most internalized form of extrinsic motivation is integrated motivation. The behavior becomes at this point fully integrated within the values, needs, and goals of an individual, resulting in a fully self-congruent pursuit of the activity. As such, an individual pursuing a goal through identified regulation enjoys the benefits they derive from their goal pursuit whereas an individual operating under integrated regulation pursues a goal because it is congruent with whom they are as a person. A last form of motivation, called amotivation, describes a lack of motivation—or intent—for a certain behavior. Consequently, goal pursuit is perceived to be fully externally controlled and without significance to the individual. Intrinsic, integrated, and identified motivations form together the overarching autonomous motivation, whereas introjected and external motivations form controlled motivation (Deci and Ryan, 1985b, 2000; Ryan and Deci, 2000a,b). Overall, amotivation and external motivation are associated with negative outcomes and autonomous motivations with positive outcomes, with introjected motivation falling somewhere in the middle (Ng et al., 2012; Taylor et al., 2014; Vasconcellos et al., 2019).

Examination of currently used perfectionism scales shows that these motivations are reflected in some of the items used. Items from the SOP subscale (HF-MPS; Hewitt and Flett, 1991), for example, refer to more self-determined forms of motivation. However, these items also imply pressure and control (e.g., “I must,” “I feel uneasy”) that is more coherent with introjected motivation. Likewise, items of the personal standards subscale of the F-MPS (Frost et al., 1990) point to more self-determined forms of motivation (i.e., standards based on oneself, importance to oneself). However, they also involve some pressure (e.g., “thoroughly competent in everything I do”), threat (e.g., item referring to the risk of ending up as a “second-rate person”), and comparison to others, which are not conductive to self-determined striving. As such, whereas PC subscales refer exclusively to controlled forms of motivation, PS subscales are grounded in both autonomous and introjected motivations.

In sum, we propose that perfectionism can be conceptualized along a hypothetical continuum of integration to the self. Individuals present, on a continuum, different motives in the pursuit of extremely stringent standards. On the same continuum, they present different degree of self-definition, that is a stable, integrated and coherent sense of self, and a corresponding healthy but realistic self-esteem—or lack thereof (Blatt and Shichman, 1983; Blatt and Blass, 1990, 1996). This view is congruent with the notion that perfectionism presents some level of identity/self-esteem disturbance as a core characteristic (Greenspon, 2000, 2008), regardless of the dimension being studied (Hewitt et al., 2017).

**Figure 1:**
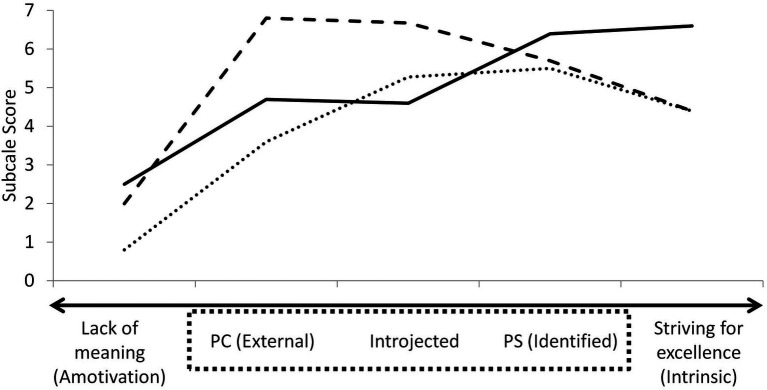
Examples of individual profiles for the Multidimensional Perfectionism and Striving for Excellence Scale.

The Motivated Perfectionism and Striving for Excellence Scale (MPSES) aims to capture both this self-definition spectrum and the motivational dimensions of the trait. This questionnaire places an ambivalent form of striving based on the absence of meaning (amotivation subscale) in opposition to a pressure-free striving for excellence (intrinsic subscale). Both poles represent the extremes of a continuum of striving, the middle of which covers all forms of extrinsic motivations for perfectionism. Specifically, the introjected subscale represents the variance overlap common to both PC and PS, whereas the external subscale is attributed solely to PC and the identified subscale to PS. The use of a dimensional approach represents a more parsimonious representation of the perfectionism trait as a motivation process, in comparison to multiple unidimensional representations, each with unique continuums of varying intensity. This approach also allows for a qualitative progression between motivations, while being intersected by gray zones representative of quantitative variations inherent to individual differences. Indeed, as the subscales are placed on a continuum, movements along this bipolar gradient denote a change from a lack of striving, to an externally motivated perfectionism, followed by a more internalized form of perfectionism, and finally to a form of striving free of pressure. However, individuals present different combinations of subscale scores along this gradient, which can be imagined as individual curves on the continuum (see Figure 1).

We chose to apply the new questionnaire to postsecondary studies specifically. It has been suggested that perfectionism is associated with different outcomes in discrete performance bursts, such as exams vs. typical daily performance (Hrabluik et al., 2012). The postsecondary academic setting comes with its own structure, providing a performance context in which a student may theoretically obtain an A+ on a class, or a 100% on a multiple choice exam. This provides a quantifiable and, sometimes, reachable threshold of “perfection,” in opposition, for example, to work as a research assistant or young clinician. It also ties in to research showing that perfectionism varies according to life domains (Dunn et al., 2005; Franche and Gaudreau, 2016) and is especially relevant for outcomes in an academic setting compared to other life domains (Levine and Milyavskaya, 2018). Finally, even though postsecondary scholastic pursuits are still marked by different forms of controls and contingencies (i.e., requirements, tests, deadlines; see Deci et al., 1991 for a review), this level of education provides more opportunities for students to be implicated in decision processes, allowing for more self-determined regulations.

The goal of the following studies was thus to create a questionnaire measuring perfectionism and striving for excellence based on a motivation continuum that represents the level of integration of the source of regulation within the self, for the postsecondary academic context. The fit of the model with the proposed dimensional structure (Study 1a) was estimated with a first sample, and convergent and divergent validity was assessed with a subset of this sample (Study 1b). Convergent and divergent validity was then assessed with an independent sample (Study 2), which also served to measure test–retest validity.

## Study 1a

The goal of this study was to create and to evaluate the fit of a new questionnaire, the MPSES. The initial step in creating the questionnaire consisted of reviewing current questionnaires relating to motivation and perfectionism. Questionnaires were scrutinized for vocabulary and content as a basis for the creation of a number of items for each type of motivation-based perfectionism and striving for excellence. Items were mostly modeled on the HF-MPS (Hewitt and Flett, 1991) and the different versions of the Self-Regulation Questionnaire (Ryan and Connell, 1989), such as the Academic Self-Regulation Questionnaire (SRQ-A; Ryan and Connell, 1989) or the learning self-regulation questionnaire (Black and Deci, 2000). As a reminder, the HF-MPS (Hewitt and Flett, 1991) measures perfectionism on three dimensions: self-oriented perfectionism (SOP), socially prescribed perfectionism (SPP), and other-oriented perfectionism (OOP). The SRQs (Ryan and Connell, 1989) measure motivation related to a specific class of behaviors as per the SDT continuum. Accordingly, regardless of the domain studied, they were useful as a resource for wording related to motivational concepts. The HF-MPS (Hewitt et al., 1991; Hewitt and Flett, 1991) and SRQ questionnaires (Ryan and Connell, 1989; Black and Deci, 2000; Levesque et al., 2006) were also both chosen for their widely supported validity and clear content.

The items created were reviewed for clarity and content before being submitted to a double back-translation process. For this, items were translated into French by one member of the laboratory and translated back into English by another member. Following this, problematic items were revised and submitted to a second translation process. Any item still proving problematic following this process was discussed until a consensus was reached. The resulting 43 items were randomized to create the initial questionnaire.

### Method

#### Participants

Sixty post-secondary students completed a paper form of the questionnaire through direct contacts. However, to facilitate recruitment, a web-version of the questionnaire was created, and was answered by 207 participants, for a total sample of 267 participants. For the web-based version of the questionnaire, a subset of participants was given the study link as well as a single use password to ascertain that participants did not participate multiple times. However, as this process proved discouraging for participants, this limitation was removed. Participants were mostly undergraduate students (52.1%) studying at the University of Quebec in Montreal (79%). Participants’ mean age was 26.64 (*SD* = 7.42) and 69.7% identified as female.

#### Procedure

Participants were informed that the questionnaire measured motivation to do well in school. They were instructed to indicate their level of agreement with each item on a seven-point Likert scale anchored with 1—*total disagreement* and 7—*total agreement* with the item. This Likert scale gradation was chosen following the HF-MPS’ (1991). The MPSES items are listed in the Supplementary material. Standard demographic questions were added at the end of the questionnaire. Data collected contained information about age, gender, nationality, study program, level of education, as well as post-secondary institution attended. Of these 267 participants, a subsample of 97 also answered questionnaires pertaining to convergent and divergent reliability (see Study 1b). No compensation was offered for participating in this study.

#### Data analysis plan

A continuum-based measure offers an interesting analysis dilemma. Continua, by their nature, posit that adjacent constructs will be related, following a simplex structure (Guttman, 1954). As such, statistically, these constructs have to show a certain level of cross-loadings. On some level these are desired as they represent the underlying relationship between the constructs. However, standard procedures, such as confirmatory factor analysis (CFA), postulate that factors are orthogonal and constrain cross-loadings to zero. Consequently, this can lead to biased model estimates, and to inflated factor correlations, as these small cross-loadings are forcefully re-expressed as higher-order correlations (Asparouhov and Muthén, 2009). Also, because of the underlying cross-loadings, the model fit tends to be mediocre. As a result, statistical support for the multidimensional structure of the measured variable and the discriminant validity of its underlying constructs tends to be unconvincing (Marsh et al., 2011). A newer statistical procedure, called exploratory structural equation modeling (ESEM), integrates the advantage of both the CFA and the exploratory factor analyses (EFA) measurement models. It allows cross-loadings and thus generates a more adequate modeling of the data. In previous studies on motivation (Marsh et al., 2011; Howard et al., 2018), the ESEM framework has produced models with a better fit, as well as with lower correlations between factors than traditional CFA analyses.

The ESEM model was assessed using Mplus 8.0 (Muthén and Muthén, 2017), using the robust maximum likelihood method (MLR) and target rotation. The syntax used followed the structure provided by Marsh et al. (2020) in their Supplementary material (see the Supplementary material of this article for the adapted syntax). Chi-square was chosen as a goodness-of-fit index, rather than a formal test index, as it is susceptible to sample size and model complexity (Schermelleh-Engel et al., 2003), both concerns with our model. It is proposed that a ratio *Χ^2^*/*df* below 3 represents an acceptable fit (Schermelleh-Engel et al., 2003). Other index measures were chosen to supplement the chi-square as further descriptive information of model fit (Kline, 2011). Standardized Root Mean Square Residual (SRMR) and Root Mean Square Error of Approximation (RMSEA) were chosen as absolute fit indexes while the Comparative Fit Index (CFI) and the Tucker-Lewis Index (TLI) were chosen as incremental fit indexes. It has been suggested that the TLI, CFI, and RMSEA are less sensitive to sample size than other indices (Hu and Bentler, 1998).

Different values have been proposed as cutoff scores, and some have become normative golden rules. One commonly cited guideline is the Hu and Bentler (1998, 1999) Monte Carlo simulation study. The authors propose that values greater than 0.95 for the TLI and CFI are indicative of a good fit, and values lower than 0.08 and 0.06 are indicative of good fit for the SRMR and RMSEA, respectively. However, these standards have been criticized as overly stringent (Marsh et al., 2004), and have not been fully replicated (Fan and Sivo, 2005). Indeed, cutoffs values may vary based on different contextual elements, such as sample size, and could be index specific, that is, nongeneralizable to an index class (Sivo et al., 2006). It follows that some researchers (Schermelleh-Engel et al., 2003; Marsh et al., 2004; Heene et al., 2011; Kline, 2011) have warned against overly strict reliance on cutoff scores and/or encouraged a more discriminate approach to these descriptors. Next to model fit, we also assessed the quasi-simplex structure of the subscales as well as their reliabilities using the program jamovi 1.1.5 (The jamovi project, 2019). We also used generalized linear modeling (GLM) to assess the effect of age, consistent with past studies having found that perfectionism can vary with age (Stoeber and Stoeber, 2009; Smith et al., 2019). Analyses of the effect of gender were exploratory.

Sample size was judged to be satisfactory for the chosen analyses. Traditional approaches have suggested that samples for structural equation modeling should be at a minimum composed of 200 subjects. Alternatively, they propose using a ratio, ideally of 20 cases per parameter (Kline, 2011). Based on these approaches, the obtained sample is on the lower end of desirable. On the other hand, newer approaches suggest sufficient sample size is to be determined on a case-by-case basis (Wolf et al., 2013). The data presented very little missing data (i.e., 0.0349% missing values). Furthermore, the initial model provided numerous indicators per factor, ranging from six for less complex subscales (e.g., amotivation), to nine for more complex subscales (e.g., external regulation), which can compensate for the smaller sample (Wolf et al., 2013).

### Results

#### Data preparation

Data were scanned for problems (e.g., using always the same answer for all questions, or creating patterns of responses) before being entered into statistical programs. No problematic data were detected. Missing data points occurred only in paper questionnaires, for a total of four occurrences on four different items. The data points were deemed to be missing at random and were left as is. No further transformations were applied.

#### Data analyses

##### Exploratory structural equation modeling

The initial model included all 43 items. Based on initial analyses, integrated and identified items were combined into one scale as it has been shown that these facets are difficult to separate through self-report scales (Vallerand et al., 1992; Howard et al., 2017). In a first step, we removed items that had little to no variance. We then removed items that cross-loaded fairly equally across the subscales. In a third step, we removed items that did not load significantly on their intended factor. Finally, we inspected correlated errors for a small number of items within a given subscale and allowed those that reflected parallel wording. For example, the error for the item “doing things less than impeccably makes me feel guilty” correlated with the error for “Making mistakes in my exams or schoolwork makes me feel guilty.” Error correlations were added in increment, from strongest to weakest, with verification of their effect on the scale fit. See Supplementary Table S1 in the Supplementary material for a list of item errors that were allowed to correlate. This procedure resulted in an adequate fit of the model (see Table 1). Items in the final model loaded significantly on their factor and had acceptable cross-loadings with other subscales. Supplementary Table S2 in the Supplementary material shows all item loadings and indicates which items were removed.

**Table 1:** Model fit, Study 1a.

Model	*Χ* ^2^	Df	*Χ*^2^/df	P	CFI	TLI	RMSEA	SRMR	Description
ESEM M0	1308.483^***^	698	1.875	291	0.891	0.859	0.057	0.036	Base model with all items (ESEM)
ESEM M1	1227.912^***^	661	1.858	284	0.897	0.866	0.057	0.035	Removal of low variance items
ESEM M2	780.749^***^	460	1.697	242	0.927	0.899	0.051	0.033	Removal of items cross-loading everywhere
ESEM M3	490.898^***^	320	1.534	207	0.953	0.932	0.045	0.029	Removal of items not loading on subscales
ESEM M4	433.638^***^	316	1.372	211	0.968	0.953	0.037	0.027	Correlated item errors within subscales

P, Parameters. ^***^p < 0.001.

##### Reliabilities

Based on the items retained in the ESEM, five subscales were created. Table 2 shows the reliabilities which range from 0.788 to 0.893 and are overall very satisfactory.

**Table 2:** Reliabilities, means, standard deviations, and intercorrelations of each subscale, Study 1a.

Subscale	M	SD	α	ω	Amotivation	P. External	P. Introjected	P. Identified	Excellence
Amotivation	2.707	1.393	0.863	0.866					
P. External	3.615	1.257	0.788	0.794	0.205^***^				
P. Introjected	4.299	1.289	0.890	0.892	0.209^***^	0.565^***^			
P. Identified	5.260	1.014	0.889	0.893	−0.100	0.344^***^	0.629^***^		
Excellence	5.117	1.213	0.884	0.887	−0.230^***^	0.103	0.329^***^	0.665^***^	
Index score	1.293	0.962			−0.770^***^	−0.256^***^	0.030	0.514^***^	0.751^***^

P, Perfectionism. ^***^p < 0.001.

##### Quasi-simplex structure

Simplex structure analyses are especially useful for continuum-based measures. This type of analysis posits that cousin constructs correlate more strongly together then more distal constructs (Guttman, 1954), thus providing evidence for a continuum of measurement. However, contrary to simplex structures, quasi-simplex structures allow for measurement errors and consequently are more reasonable representations of psychological measures (Jöreskog, 1970), and more specifically of the SDT continuum (Litalien et al., 2017). Since first proposed by Ryan and Connell (1989), a quasi-complex structure has been quite consistently supported for the SDT continuum, as shown by a meta-analysis by Howard et al. (2017).

Results (see Table 2) showed that amotivation correlated weakly with the other subscales, apart from the identified subscale. Notably, it correlated negatively with the striving for excellence subscale. External perfectionism correlated with all other perfectionism subscales, and most strongly with introjected perfectionism. Introjected perfectionism correlated more strongly with adjacent subscales than more distal ones. Identified perfectionism adhered to the expected pattern of relationships; it correlated with the other perfectionism subscales, and more strongly with striving for excellence.

The total score for the scale needs to reflect the different weightings based on the underlying dimensionality of the scale. One frequent approach is the relative autonomy index, which consists of a weighed sum, in which each subscale is assigned a weight corresponding to its placement on the continuum (Grolnick and Ryan, 1987, 1989). More controlled forms of motivation are given a negative weight and more autonomous forms of motivation are given a positive weight (see Sheldon et al., 2017, for a review of scoring procedures). For example, external motivation is given a weight of −2 and introjected motivation a weight of −1, while identified and intrinsic motivations are given a weight of 1 and 2, respectively, (Fortier et al., 2011; Kusurkar et al., 2013; Vancampfort et al., 2015). Others (Vallerand and Bissonnette, 1992) have also included the amotivation and the integrated subscales, leading to a weighted continuum of −3 to +3.

Conversely, our own theoretical framework suggests that the index score should not exactly follow this frequently used formula. Indeed, consistent with the meta-analysis of Howard et al. (2017), which showed introjected regulation to be equidistant to external and identified regulation; introjected perfectionism represents the middle point of our hypothesized continuum. Consequently, we calculated the index score with introjected perfectionism as a middle point (0), with lack of striving (i.e., amotivation) and external perfectionism as negatively weighted subscales, and identified perfectionism and striving for excellence (i.e., intrinsic motivation) as positively weighted subscales. Furthermore, as our subscales did not have an equal number of items, we used a mean weighted score instead of a sum weighted score. It follows that in future research, both the use of the individual subscales scores and the use of the index score could serve different research designs and be pertinent in assessing different outcomes.

Using the created index score as a dependent variable, we conducted a GLM analysis with age and gender as a covariate and a factor, respectively. The resulting model was non-significant (see Supplementary material for details).

### Interim discussion

The first goal of this study was to create a scale measuring perfectionism and striving for excellence from a motivational perspective, both underlain by an implicit degree of self-definition. ESEM proved to be a good fitting model and provided support for our framework, which joins all construct through an underlying continuum of integration. Based on the ESEM analysis, a scale composed of 31 items was retained.

Notably, the analyses revealed that the final external perfectionism subscale referred solely to interpersonal rewards and pressures. Even though grades were theorized to be an external reward and initially included, these items instead loaded on identified perfectionism and/or introjected perfectionism. Further consideration of the importance of grades led us to conclude that grades can also be considered an indicator of performance and not only an external rewarding or punishing outcome. In turn, the value of this indicator is contingent on the source of the standard being applied. For example, a grade can fulfill a self-esteem need or serve as a platform to go into higher education. Thus, these items were poor indicators of any specific form of motivation and excluded from our model. Likewise, items implying pressure without an accompanying qualifier were rejected. Reaching the highest performance possible for instrumental purposes necessarily implies some level of pressure and it is not the mere presence of felt pressure but rather the underlying source of this pressure that differentiates dimensions of perfectionism. Finally, amotivation items denying any form of striving were rejected, whereas items reflecting a lack of meaning were retained, as a result of both statistical analyses and theoretical scrutiny. As we propose a continuum underlain by both motivational processes and identity integration, a lack of meaning is more coherent than an absence of striving. Furthermore, to know that one is not a perfectionist and does not strive for excellence reflects a realistic evaluation and knowledge of oneself that would be incoherent with the leftmost end of the continuum, as it represents a severe lack of self-definition.

The new subscales showed adequate fit, had acceptable reliabilities and followed a quasi-simplex structure. These analyses thus provided support for the structure of our questionnaire. A second goal of this study was to assess convergent and divergent validity. For this purpose, a subsample of 97 participants answered supplementary questionnaires.

## Study 1b

As the MPSES focuses on academic achievement, we assessed its relation to constructs relevant to this domain. However, we used generalized scales for the motivation and perfectionism constructs to reduce capitalizing on shared language. First, measures of general perfectionism and goal orientation were used to assess if subscales converged with expected general trait orientation for perfectionism and for motivation. A scale that measures achievement attitudes and behaviors (Waugh, 2002) was thought to be useful for further differentiation of the perfectionism subscales, as well as to differentiate perfectionism from striving for excellence. It assesses intensity of behaviors in setting goals and in pursuing these goals through measuring the frequency with which they occur in students’ scholastic pursuits. The scale assesses facets such as setting standards and efforts, representative of intensity of striving, which has been suggested to be characteristic to perfectionism. It also assesses goals and tasks, which measures behaviors and attitudes pertaining to choosing difficult but reachable goals and tasks. This measured approach to goal setting has been suggested to be at odd with the excessive (Gaudreau, 2019) and unrealistic standards (Hewitt et al., 2017) underlining perfectionism. Finally, it also presents subscales called intrinsic rewards and interest, both attributed to intrinsic motivation in the SDT literature (Deci and Ryan, 1985b, 2000; Ryan and Deci, 2000a).

Furthermore, consistent with Hewitt and Flett (1991) flagship scale creation article, participants were asked about their minimal and ideal grade standard, and their environment’s minimal and ideal grade standard. These questions reflect the perceived origin of the standards (e.g., others or within the self) for extrinsic motivations, and assess the difference between lowest acceptable performance, an avoidance performance goal, and ideal performance, an approach performance goal. Moreover, participants were asked how important it was for them to do well, and to reach their own goals and standards, as well as the goals and standards that others hold for them.

A measure of personality dispositions was used to further assess convergent validity. A recent meta-analysis by Smith et al. (2019) showed that subscales related to PC are positively associated with neuroticism and negatively with conscientiousness. On the other hand, subscales related to PS are associated with conscientiousness and weakly with neuroticism. Likewise, more autonomous forms of motivation have been associated with conscientiousness. However, more controlled forms of motivation have shown inconsistent relations with neuroticism and conscientiousness (Ingledew et al., 2004; Komarraju et al., 2009; Clark and Schroth, 2010). A similar profile of results was expected for the perfectionism subscales and striving for excellence. More specifically, we expected external perfectionism to correlate solely with neuroticism and identified perfectionism as well as striving for excellence to correlate solely with conscientiousness. Introjected perfectionism was expected to correlate with both traits.

Self-derogation, as a construct related to self-definition, was also used to assess validity. Based on studies assessing cousin constructs (Gilbert et al., 2006; Dunkley et al., 2012; Linnett and Kibowski, 2019), self-derogation was thought to correlate with external and introjected perfectionism but not with the striving for excellence subscale. Finally, considering the importance of self-presentation for perfectionistic individuals (Hewitt et al., 2003; Mackinnon and Sherry, 2012; Nepon et al., 2016), a measure of social desirability was used to verify participants’ answers had not been overly influenced by such concerns.

### Method

#### Participants

The sample of 97 participants was a subsample of the Study 1a sample. An *a priori* power analysis showed that a sample of 84 individuals was required to detect a medium effect size with a power of 0.80 and an alpha of 0.05. As such, the sample acquired is sufficient to detect small to medium effect sizes, but cannot detect very small effects. Data collection continued until the end of the term. Sixty-seven percent of participants studied at UQAM. Participants were mostly bachelor students (59.8%) from various study programs (32% psychology). Mean age was 27.58 year (*SD* = 8.32) and 77.3% of participants identified as female.

Participants were invited to complete the MPSES, as well as a series of other scales as online questionnaires. Participants completed the HF-MPS (Hewitt and Flett, 1991; Labrecque et al., 1999), the General Causality Orientation Scale (GCOS; Deci and Ryan, 1985a; Vallerand et al., 1987), the short version of the Marlowe-Crowne (Crowne and Marlowe, 1960; Blais et al., 1991), the Self-Derogation Scale (Kaplan and Pokorny, 1969), the NEO Five Factor Inventory (NEO-FFI; Costa and McCrae, 1992; Sabourin and Lussier, 1992), and the scale of Motivation to Achieve Academically (MAA; Waugh, 2002). They also answered questions about academic standards extracted from validation studies of Hewitt and Flett (1991) for the HF-MPS, as well as demographics related questions. Questions about academic standards taken from Hewitt and Flett (1991) as well as the Motivation to Achieve Academically scale (Waugh, 2002) were back translated as no French translations were found. Table 3 provides details of each scale, along with sample items. Reliabilities for the scales were acceptable (Supplementary Table S3 in the Supplementary material).

**Table 3:** Questionnaires characteristics, Study 1b.

Questionnaire	Scoring	Sample item
HF-MPS	Likert; 1 (*totally disagree*) to 7 (*totally agree*)	Self-Oriented: I must work to my full potential at all times. Socially Prescribed: Others will like me even if I do not excel at everything. (R) Other-Oriented: I cannot stand to see people close to me make mistakes.
Standards	Letter grade Likert; 0 (*not at all important*) to 6 (*very important*)	Lowest acceptable grade- others: What is the lowest letter grade you could get that some person who is important to you would be satisfied with? Importance: How important is it to you to do well in your courses?
Attitudes Toward Academic Achievement	Frequency; 0 (*none or only one of my subjects*) to 3 (*in all or nearly all my subjects*)	Goals: I set myself realistic but challenging academic goals. Intrinsic Rewards: I like the intellectual challenge of academic work. Efforts: I make strong demands on myself to achieve in academic work.
GCOS	Likert; 1 (*very unlikely*) to 7 (*very likely*)	Sample vignette: You are embarking on a new career. The most important consideration is likely to be: Impersonal: Whether you can do the work without getting over your head. Autonomy: How interested you are in that kind of work. Control: Whether there are good possibilities for advancement.
NEO-FFI	Likert; SD (*strongly disagree*) to SA (*strongly agree*)	Openness: I do not like to waste my time daydreaming. (R) Conscientiousness: I keep my belongings neat and clean. Extraversion: I like to have a lot of people around me. Agreeableness: I try to be courteous to everyone I meet. Neuroticism: I am not a worrier. (R)
Social Desirability	True or false	I am always willing to admit it when I made a mistake.
Self-Derogation	Likert; *Strongly disagree* to S*trongly agree*	At times, I think I am no good at all.

(R) denotes items which are reverse-coded.

#### Material

##### Hewitt and Flett multidimensional perfectionism scale

The HF-MPS (Hewitt and Flett, 1991) is composed of 45 items and measures perfectionism based on manifestations related to the self and social dimensions, that is self-oriented perfectionism, socially prescribed perfectionism, and other-oriented perfectionism. The HF-MPS (Hewitt and Flett, 1991) has been widely used in the literature and shown to be quite reliable. The French adaptation of the HF-MPS (Labrecque et al., 1999) has been validated with students, community, and clinical samples and has acceptable psychometric parameters (Labrecque et al., 1999; Miquelon et al., 2005).

##### Academic standards

Questions pertaining to academic standards were taken from validation study of Hewitt and Flett (1991). Four items pertained to minimal and ideal scholastic results, two items to the importance of reaching goals and standards, and one item to the importance of doing well. All questions were divided on a bipolar self vs. other dimension, apart from the one item about doing well.

##### Motivation to achieve academically scale

The MAAS (Waugh, 2002) measures motivation as attitudes toward accomplishment as evidenced by behaviors. Subscales include Standards, Goals, Tasks, Effort, Values, Interest, Learning from Others, Responsibility for Learning, Intrinsic Rewards, and Social Rewards. The measure is composed of 24 items and participants are required to indicate both what they aim to do and what they truly do in their scholastic pursuits.

##### General causality orientation scale

The GCOS (Deci and Ryan, 1985a) measures initiation and regulation of behaviors as per individuals’ perceived causality. These causality orientations are considered stable tendencies akin to personality traits. Autonomy orientation is defined by a sense of choice in producing a behavior and relates to an internal perceived locus of causality. Control orientation is defined by a perception that behaviors result from an internal or external control. It is characterized by a feeling of pressure, which is reminiscent of an external perceived locus of causality. Finally, impersonal orientation is defined by the perception that an individual’s behavior is out of their control. Such an orientation manifests through a perceived incapacity to regulate one’s behavior toward a particular result. Therefore, desired outcomes are seen as beyond control as a result of internal (e.g., incompetence) and external (e.g., task difficulty) forces (Deci and Ryan, 1985a). The scale has been shown to have acceptable psychometric properties (Deci and Ryan, 1985a) and the French version of the scale showed acceptable internal consistency (Vallerand et al., 1987). It is composed of 12 vignette scenarios for which each orientation is assessed.

##### NEO five factor inventory

The NEO-FFI (McCrae and Costa, 1992) is a short version of the NEO-PI-R, with 60 items out of the original 240. The scale measures the five big personality traits, which are agreeableness, conscientiousness, extraversion, openness to experience, and neuroticism. The shorter scale was chosen due to time limitation. It allows an adequately reliable measurement of the big five traits (Caruso, 2000; McCrae and Costa, 2004). A French version translated by Sabourin and Lussier (1992) was chosen. The translation showed good psychometrics.

##### Marlowe-Crowne social desirability scale

The short 10 items French version by Blais et al. (1991) of the Marlowe-Crowne scale (Strahan and Gerbasi, 1972) was used to measure social desirability.

##### Self-derogation

The Self-Derogation scale (Kaplan and Pokorny, 1969) is based on a factor analysis of the Rosenberg Self-Esteem Scale (Rosenberg, 1965). The scale measures global negative self-attitudes or affects about oneself. The Self-Derogation Scale contains the seven items of the Rosenberg Self-Esteem Scale (RSES; Rosenberg, 1965) that were regrouped under the first factor. Scores on positive items must be reversed (Kaplan and Pokorny, 1969). Since the RSES (Rosenberg, 1965) had already been translated in French and validated, the corresponding items were chosen from a published translation by Vallières and Vallerand (1990).

### Results

#### The MPSES subscales

Data was scanned for problems, as in Study 1a. No problems were detected. Missing data occurred as a result of a programming issue for five items on the MAA scale for a maximum of five data points per item. No further transformations were applied. Results of correlation analyses between the MPSES, the HF-MPS, and the GCOS are presented in Table 4.

**Table 4:** Correlations between the MPSES and the HF-MPS and GOCS, Study 1b.

Subscale	SOP	SPP	OOP	AO	CO	IO
Amotivation	−0.068	0.205^*^	−0.125	−0.151	0.048	0.352^***^
P. External	0.470^***^	0.695^***^	0.240^*^	−0.105	0.168	0.280^**^
P. Introjected	0.751^***^	0.509^***^	0.202^*^	−0.020	0.208^*^	0.251^*^
P. Identified	0.609^***^	0.153	0.211^*^	0.170	0.242^*^	0.005
Excellence	0.328^**^	−0.045	0.144	0.245^*^	0.214^*^	−0.107
Index	0.186	−0.291^**^	0.114	0.261^*^	0.079	−0.318^**^

P., Perfectionism; SOP, Self-Oriented Perfectionism; SPP, Socially Prescribed Perfectionism; OOP, Other-Oriented Perfectionism; AO, Autonomous Orientation; CO, Controlled Orientation; IO, Impersonal Orientation. ^*^p < 0.05, ^**^p < 0.01, and ^***^p < 0.001.

We predicted that amotivation and external perfectionism correlate with SPP and that identified perfectionism and striving for excellence correlate with SOP. We also predicted that introjected perfectionism is related to both HF-MPS subscales.

We found that external and introjected perfectionism, as well as amotivation, correlated with SPP. Self-oriented perfectionism correlated with all perfectionism subscales and with striving for excellence. Interestingly, it correlated the strongest with introjected perfectionism. The external, introjected, and identified perfectionism subscales correlated with OOP, though weakly. Partialling correlations for the overlap between SOP and SPP (see Supplementary Table S4 in the Supplementary material) merely changed the strength of the association between these subscales and the MPSES, apart from SPP, which now correlated weakly with striving for excellence. As such, the results overall followed our predicted outcomes, except for the correlation between SOP and external perfectionism.

For the GCOS, we predicted that amotivation and external perfectionism correlate with impersonal orientation. We also predicted that all forms of perfectionism correlate with control orientation. Finally, we predicted that identified perfectionism and striving for excellence correlate with an autonomy orientation. Results for the GCOS indicated that only striving for excellence correlated positively and significantly with the autonomous orientation. Control orientation correlated positively with introjected perfectionism as well as with identified perfectionism and striving for excellence. Further analyses decomposing the control orientation subscale showed only a few items drove the relation with identified perfectionism and striving for excellence. These items referred to doing assignments, possibilities for advancement, and feeling excited about status and salary. Finally, impersonal orientation correlated with amotivation, as well as with external and introjected perfectionism. Examination of items driving the correlation with introjected perfectionism revealed that they referred to ego threats and self-judgments, such as “work without getting in over your head,” “not good enough for the job,” and “cannot do anything right.” In sum, most of our hypotheses were confirmed. Yet, two results were unexpected: external perfectionism only correlated with impersonal orientation and identified perfectionism did not correlate with autonomy orientation. These results may be explained by the smaller than ideal sample size. However, it is also possible that external perfectionism is solely defined by the perception that the desired standards are simply out of reach. Likewise, identified perfectionism could solely be defined by inner controls and instrumental contingencies, and therefore lacking in pressure free choice making and striving.

For questions relating to the importance of achieving goals and standards, our predictions reflected the self/other dimensions of the MPSES. We thus expected introjected perfectionism to be correlated with both self and others’ goals and standards, whereas external perfectionism was expected to correlate only with others’ goals and standards and identified perfectionism and striving for excellence with self-originating goals and standards. Concerning minimal and ideal grades, we expected external perfectionism to correlate with the minimal performance standard and striving for excellence to correlate with the ideal performance standard. Introjected perfectionism and identified perfectionism were expected to correlate with both minimal and ideal performance. Finally, we expected all forms of perfectionism and striving for excellence to correlate to some degree with the importance of doing well, but more so for introjected and identified perfectionism. As amotivation relates to a lack of meaningful striving, we predicted that correlations with this subscale are largely nonsignificant. Results are presented in Table 5.

**Table 5:** Correlations between the MPSES and Questions About Performance, Study 1b.

Subscale	DW	LgS	LgO	IgS	IgO	RgS	RgO
Amotivation	−0.028	0.026	−0.014	−0.133	−0.002	−0.199	0.236^*^
P. External	0.248^*^	0.153	0.140	−0.015	0.127	−0.040	0.617^***^
P. Introjected	0.426^***^	0.220^*^	−0.023	0.109	0.021	0.200^*^	0.307^**^
P. Identified	0.599^***^	0.324^**^	−0.083	0.291^**^	0.081	0.525^***^	0.054
Excellence	0.381^***^	0.242^*^	−0.128	0.252^*^	0.102	0.454^***^	−0.168
Index	0.248^**^	0.124	−0.108	0.254^*^	0.032	0.440^***^	−0.366^***^

P, Perfectionism; DW, Importance of doing well; LgS, Lowest acceptable grade for oneself; LgO, Others’ lowest acceptable grade; IgS, Ideal grade for oneself; IgO, Others’ ideal grade; RgS, Importance of reaching one’s goals and standards; and RgO, Importance of reaching others’ goals and standards. ^*^p < 0.05, ^**^p < 0.01, ^***^p < 0.001.

Analyses showed a significant relationship between the importance of doing well in school and all perfectionism subscales and striving for excellence. Amotivation and external perfectionism correlated positively with the importance of reaching others’ goals and standards, whereas identified perfectionism and striving for excellence correlated positively with the importance of reaching one’s own goals and standards. Introjected perfectionism correlated with both the importance of reaching one’s and others’ goals and standards. Minimal performance correlated with introjected and identified perfectionism, and with striving for excellence only for the self-originating standard. Likewise, only the self-originating ideal performance correlated with identified perfectionism and striving for excellence.

Even though many results went in the expected directions, some were mixed. Amotivation did correlate with the importance of reaching others’ goals and standards. It is noteworthy that other’s minimal and ideal performance standard did not correlate with any subscale, whereas self-originating minimal and ideal performance standards both correlated with identified perfectionism and striving for excellence.

Table 6 shows correlations of the MPSES subscales with the MAA scale. Overall, we expected that, as the standard become more internalized, behaviors and attitudes toward achievement appear more frequently. Operationally, this meant more significant correlations and stronger correlations for striving for excellence than identified perfectionism, for identified perfectionism than introjected perfectionism, etc. However, we also expected certain specific results, such as striving for excellence being correlated with interest and intrinsic rewards, more so than the perfectionism subscales. We also only expected identified perfectionism and striving for excellence to be correlated with the goals and tasks subscales. As they both refer to choosing hard but doable goals and tasks, they implicitly reflect a perception of one’s capacities to succeed in reaching goals and completing tasks.

**Table 6:** Correlations between the MPSES and the Motivation to Achieve Academically Scale, Study 1b.

	Standards		Goals		Tasks		Efforts		Value	
Subscale	Aim	Do	Aim	Do	Aim	Do	Aim	Do	Aim	Do
Amotivation	−0.131	−0.097	0.007	−0.077	−0.239^*^	−0.200^*^	−0.117	−0.189	−0.066	0.020
P. External	0.072	−0.027	0.074	0.078	0.068	0.014	0.081	0.000	0.153	0.209^*^
P. Introjected	0.202^*^	0.098	0.023	0.113	0.075	0.113	0.126	0.117	−0.037	0.002
P. Identified	0.401^***^	0.368^***^	0.141	0.378^***^	0.226^*^	0.328^**^	0.284^**^	0.431^***^	−0.036	0.026
Excellence	0.297^**^	0.261^*^	0.236^*^	0.451^***^	0.340^***^	0.471^***^	0.257^*^	0.353^***^	0.019	0.089
Index	0.272^**^	0.253^*^	0.118	0.312^**^	0.320^**^	0.396^***^	0.221^*^	0.357^***^	−0.003	−0.020
	**Interest**		**Peers**		**Responsibility**		**Intrinsic Rewards**		**Social Rewards**	
**Subscale**	**Aim**	**Do**	**Aim**	**Do**	**Aim**	**Do**	**Aim**	**Do**	**Aim**	**Do**
Amotivation	−0.102	−0.154	−0.174	−0.220^*^	−0.186	−0.240^*^	−0.163	−0.190	−0.068	−0.140
P. External	0.104	−0.029	0.087	−0.071	0.036	−0.015	−0.147	−0.178	0.108	0.067
P. Introjected	0.034	0.072	−0.120	−0.170	−0.057	0.010	−0.228^*^	−0.129	−0.058	−0.008
P. Identified	0.192	0.372^***^	0.069	0.125	0.208^*^	0.398^***^	0.112	0.327^**^	0.088	0.204^*^
Excellence	0.271^**^	0.445^***^	0.109	0.276^**^	0.301^**^	0.498^***^	0.399^***^	0.505^***^	0.140	0.290^**^
Index	0.196	0.379^***^	0.137	0.296^**^	0.278^**^	0.453^***^	0.343^**^	0.460^***^	0.093	0.238^*^

P, Perfectionism. ^*^p < 0.05, ^**^p < 0.01, and ^***^p < 0.001.

Amotivation was negatively correlated with several subscales. Specifically, it was negatively correlated to “doing” subscales, such as choosing tasks to succeed and responsibility for one’s learning. This is in line with the lack of meaningful striving that defines amotivation. Both external and introjected perfectionism did not correlate significantly with subscales indicative of active striving toward achievement. The external perfectionism subscale only correlated with the value subscale, which referenced rethinking one’s values when facing time conflicts, and the introjected perfectionism subscale only correlated positively with the aiming section of the standards subscale (i.e., aiming to do one’s best to reach one’s high standards and evaluating one’s performance against these standards). On the other hand, the identified perfectionism subscale and the striving for excellence subscale correlated with numerous subscales, both as an aim and as a behavior.

Of interest, the goals and tasks subscales correlated with both identified perfectionism and striving for excellence. Decomposing the task subscale showed that the identified perfectionism and striving for excellence subscales only correlated with items referring to seeking difficult tasks, and not the item referring to seeking average tasks. These results suggest that identified perfectionism and striving for excellence are associated with seeking out opportunities to test oneself in challenging ways. However, results also showed that, comparatively, identified perfectionism was more strongly associated with subscales indicative of striving to reach a desired level of performance (i.e., standards and efforts), whereas striving for excellence was more strongly associated with subscales indicative of enjoying the striving process (i.e., interest and rewards). In the same vein, striving for excellence correlated more strongly with subscales indicative of a desire to learn, such as taking responsibility for learning and seeking knowledge from peers and experts. Comparatively, SPP correlated negatively with the intrinsic rewards subscale as an aim, *r*(94) = −0.244, *p* = 0.016, and as a behavior, *r*(94) = −0.232, *p* = 0.022. SOP only correlated with standards, as an aim, *r*(94) = 0.311, *p* = 0.002, and as a behavior, *r*(90) = 0.262, *p* = 0.011, and efforts as an aim, *r*(94) = 0.243, *p* = 0.016 and as a behavior, *r*(94) = 0.260, *p* = 0.010. These results suggest that the MPSES’s specificity is able to tap into more behaviors and attitudes that differentiate PS and PC than the HF-MPS.

Overall, the results of the correlation analyses followed our expectations. Nonetheless, the degree to which external perfectionism and introjected perfectionism were found to not correlate with active pursuit of the standard was unexpected. Indeed, it suggests that even though these subscales might be associated with the perception that one holds and pursue perfectionistic standards, in these cases, intentions do not translate into attitudes or actions.

Our final analyses concerned the relationship between the MPSES and the NEO-FFI, as well as the Self-Derogation scale and the Marlowe-Crowne Social Desirability Scale, the results of which are presented in Table 7. Most importantly, we expected external and introjected perfectionism to be positively correlated with neuroticism and self-derogation. We also predicted that striving for excellence is negatively correlated with neuroticism and self-derogation, and positively correlated with conscientiousness. Finally, we predicted a positive relation between identified perfectionism and conscientiousness.

**Table 7:** Correlations between the MPSES and the NEO-FFI-R, Self-Derogation Scale and Social Desirability Scale, Study 1b.

Subscale	O	C	E	A	N	SD	MC
Amotivation	0.022	−0.361^***^	−0.241^*^	−0.089	0.376^***^	0.371^***^	−0.183
P. External	−0.078	−0.013	−0.091	−0.126	0.483^***^	0.494^***^	−0.079
P. Introjected	−0.191	0.217^*^	−0.110	−0.217^*^	0.436^***^	0.329^**^	0.057
P. Identified	−0.026	0.607^***^	0.127	−0.100	0.026	−0.137	0.087
Excellence	0.037	0.513^***^	0.181	−0.154	−0.229^*^	−0.266^**^	0.208^*^
Index	0.022	0.565^***^	0.268^**^	−0.012	−0.441^***^	−0.490^***^	0.238^*^

P., Perfectionism; O, Openness; C, Conscientiousness; E, Extraversion; A, Agreeableness; N, Neuroticism; SD, Self-Derogation; and MC, Marlowe-Crowne Social Desirability. ^*^p < 0.05, ^**^p < 0.01, and ^***^p < 0.001.

Amotivation, external perfectionism, and introjected perfectionism correlated positively with neuroticism and self-derogation, whereas striving for excellence correlated negatively with both of these subscales. Conscientiousness correlated positively with introjected perfectionism, albeit weakly, as well as with identified perfectionism and striving for excellence. A close look revealed the item “I strive for excellence in everything I do” strongly drove the effect for introjected perfectionism. Other items included words such as “perform” and “getting the job done.” Amotivation correlated negatively with conscientiousness and extraversion, and introjected perfectionism correlated negatively with agreeableness. Overall, results confirmed our hypotheses but also suggested that social desirability correlated positively with intrinsic motivation. Further examination indicated that this result was driven by three items (i.e., *r* between 0.204 and 0.240). As such, these significant correlations were low and not systematic.

#### The composite index

The composite index of the MPSES also followed the expected pattern of results. It correlated positively with (sub)scales indicative of a higher internalization and endorsement of standards, such as the importance of doing well, the importance of reaching one’s goals and standards and the self-generated ideal grade standard. Notably, controlling for the overlap with SPP allowed the correlation between SOP and the index to become significant, *r*(94) = 0.385, *p* < 0.001. Furthermore, the index correlated negatively with variables indicative of a lower internalization of the standards, such as SPP, partial *r*(94) = −0.439, *p* < 0.001, and the importance of reaching others’ goals and standards. In the same order, the index correlated positively with autonomy orientation and negatively with impersonal orientation. Furthermore, the continuum index correlated positively with numerous MAA subscales, such as intrinsic rewards, interest, standards, tasks, efforts, and responsibility for learning. It also correlated positively with conscientiousness and negatively with self-derogation and neuroticism. Finally, it correlated positively with social desirability, but this result was driven by one item (i.e., There have been occasions when I felt like smashing things). These results thus suggest that the index score represents adequately the proposed continuum of self-determined perfectionistic strivings.

### Discussion

Study 1b shows that the MPSES is overall related to relevant constructs in the expected ways, both at the subscale level and with the full scale. However, a few low and non-significant correlations with the GCOS raised questions about our ability to detect smaller effect sizes with the sample acquired. Nonetheless, overall the results suggest that the scale measures the intended constructs of perfectionism and of striving for excellence. As a final step, we developed a short version of the scale. A new sample of participants answered the short version of the scale twice, at a 6 weeks interval, in order to assess test–retest reliability.

## Study 2

The previous analyses supported the proposed factor structure. Study 2 aimed to confirm the basic structure of the proposed model, to examine the basic correlation matrix of the scale with the big five traits of personality and a self-esteem measure, and to evaluate the test–retest validity of the scale. For this, a short version of the scale was created (see method section). Participants also answered a short measure of broad personality dispositions and of self-esteem, in order to obtain further support for the structure of the personality scale. Reliabilities for the scales were acceptable (Supplementary Table S3 in the Supplementary material).

### Method

#### Participants

The goal of this study was to evaluate the correlation matrix and the test–retest validity of the MPSES. As such, a sample of 287 post-secondary students was recruited through ads, classroom projects, and direct contact to complete a series of questionnaires online. Although a sample of 200 was first targeted, participants were allowed to take part in the study until the end of the term. Three participants were excluded from the dataset as they fell short of age of consent. Of the 287 participants, 145 answered the shortened MPSES at time 2. Participants were mostly bachelor level (54.9%) University of Quebec in Abitibi-Témiscamingue students (59%) from various study programs (9.7% psychology). Mean age of participants was 27.87 year (*SD* = 8.93) and 82.3% of participants identified as female.

Students were contacted to answer the MPSES a second time after a delay of 6 weeks had passed. In the event they did not answer following the first prompt, they were again contacted a week later as a reminder to complete their participation. Data were matched through a code created by participants at the end of the first participation. No compensation was offered in exchange for participating in the study.

#### Material

##### Big five inventory-15

The Big Five Inventory (John et al., 2008) measures the broad category traits extraversion, agreeableness, conscientiousness, neuroticism, and openness to experience on 44 items. This self-report scale measures each trait anchored in three seven-Likert-point items. The BFI-15 is a short version of the BFI-44 that has been used in German, Australian and British national surveys (Lang et al., 2011). As there is a validated translated version of the BFI-44 by Plaisant et al. (2010), we used the 15 items from this test to create a French version of the BFI-15.

##### Motivated perfectionism and striving for excellence scale

The short version of the MPSES was used to measure self-determined perfectionistic strivings. To create a short version of the MPSES we inspected the results of the reliability analyses and removed items the removal of which did not reduce reliability below the desired level of 0.75, while assuring that the scale construct was adequately covered. This resulted in a 27-item scale. Table 8 shows the correlations between full subscales and the shortened subscales based on the data from study 1a. All correlations were above 0.9, suggesting that the short scale adequately represents the full scale.

**Table 8:** Correlations between the Full Scale (Study 1a) and the Shortened Scale (Study 2).

Subscale	R
Amotivation	1.000^***^
P. External	1.000^***^
P. Introjected	0.993^***^
P. Identified	0.985^***^
Excellence	0.984^***^
Index	0.995^***^

P, Perfectionism.

##### Self-esteem

Self-esteem was measured through a single item from the Rosenberg Self-Esteem Scale (Rosenberg, 1965). The item, “On the whole, I am satisfied with myself” was measured on a four-point Likert scale; from strongly disagree to strongly agree. The item was taken from a previously validated version of the complete scale, translated by Vallières and Vallerand (1990).

##### Demographic data

Participants were also asked to provide information pertaining to their gender, age, ethnicity, study program and level, as well as university or college.

### Results

#### Data analysis plan

Data were scanned for problems, as in Study 1. No problems were detected. There were a few missing data points (i.e., nine) scattered through six items over both sampling times. No further transformation was applied.

We first assessed the model fit for the short scale for the complete sample using ESEM, *Χ^2^* (226) = 508.675, *p* < 0.001, CFI = 0.939, TLI = 0.906, RMSEA = 0.066, SRMR = 0.030 (see Table 9 for item loadings). One item did not load significantly on its factor, but was preserved to cover the identified concept, as it refers to the importance of goals. Prior to testing for invariance, we also assessed the model for both sampling times in the test re-test sample. The model fit was adequate for both Time 1, *Χ^2^* (226) = 379.457, *p* < 0.001, CFI = 0.934, TLI = 0.898, RMSEA = 0.069, SRMR = 0.036, and Time 2 data, *Χ^2^* (226) = 365.305, *p* < 0.001, CFI = 0.943, TLI = 0.912, RMSEA = 0.066, SRMR = 0.031. To test for time invariance, we used the syntax proposed by Marsh et al. (2009) which consists of 13 steps (see Table 10). We followed the procedure outlined by Marsh et al. (2010) in assessing configural invariance (model 1), weak invariance (model 1 vs. model 2), strong measurement invariance (model 2 vs. model 5), strict measurement invariance (model 5 vs. model 7), factor variance–covariance invariance (model 2 vs. model 4), and finally time mean invariance (any pairs of: model 5 vs. model 10, model 7 vs. model 11, model 8 vs. model 12, or model 9 vs. model 13). Following recommendations by Chen (2007), we used a change of 0.005 in CFI, and of 0.01 in RMSEA as criteria. The criterion for change in the SRMR was of 0.025 for factor loading and of 0.005 for intercept and residual invariance.

**Table 9:** ESEM items loadings, Study 2.

Item	Amotivation	P. External	P. Introjected	P. Identified	Excellence
Amo31	**1.271**	0.081	−0.032	−0.015	−0.010
Amo36	**1.538**	0.023	**−0.182**	−0.051	**0.189**
Amo38	**1.366**	−0.094	0.133	0.077	**−0.137**
Ext1	−0.044	**0.872**	0.243	−0.086	−0.023
Ext19	0.021	**0.827**	0.097	**0.703**	**−0.226**
Ext21	−0.072	**1.199**	0.021	**−0.207**	**0.213**
Ext24	0.058	**1.009**	0.123	−0.130	−0.041
Ext27	0.109	**1.631**	−0.095	0.105	−0.120
Intro3	−0.106	−0.026	**1.014**	**0.253**	0.013
Intro12	−0.088	0.098	**1.151**	−0.045	**0.236**
Intro20	0.157	**0.302**	**0.973**	0.076	−0.143
Intro28	0.044	**0.292**	**0.979**	**−0.384**	0.148
Intro33	0.047	0.041	**0.970**	**0.303**	−0.032
Intro40	**0.173**	0.190	**0.987**	**0.248**	−0.185
Intro42	0.102	0.131	**1.144**	**0.272**	−0.055
Ident18	−0.049	0.103	**−0.207**	**0.956**	**0.220**
Ident32	**−0.122**	0.092	−0.161	**0.83**	0.066
Ident34	−0.083	**0.176**	−0.089	**0.885**	**0.285**
Ident39	0.012	**0.355**	**0.465**	0.241	**0.316**
Integ5	−0.017	−0.135	**0.488**	**0.781**	**0.279**
Integ14	0.100	0.105	**0.539**	**0.62**	**0.275**
Integ16	0.008	−0.081	**0.403**	**0.844**	**0.423**
Integ23	**0.139**	0.064	**0.429**	**0.748**	0.166
Intrin8	0.045	−0.044	0.025	0.151	**1.198**
Intrin13	−0.004	0.084	**−0.194**	0.056	**1.551**
Intrin22	0.015	−0.146	**0.229**	**0.302**	**0.951**
Intrin29	−0.087	0.036	−0.117	**0.802**	**0.283**

P., Perfectionism. Significant weights are in bold.

**Table 10:** ESEM time invariance analyses, Study 2.

Model	Χ^2^ (df)	CFI	TLI	RMSEA	SRMR
ESEM (N = 287)	508.675^***^ (226)	0.939	0.906	0.066	0.030
M1 – Configural	1851.161^***^ (1129)	0.866	0.831	0.067	0.043
M2 – FL	1959.250^***^ (1239)	0.867	0.846	0.064	0.053
M3 – FL, Uniq	2032.984^***^ (1266)	0.858	0.840	0.065	0.058
M4 – FL, FVCV	1998.872^***^ (1254)	0.862	0.843	0.065	0.058
M5 – FL, Inter	1979.105^***^ (1261)	0.867	0.849	0.063	0.053
M6 – FL, Uniq, FVCV	2666.263^***^ (1281)	0.744	0.714	0.087	0.061
M7 – FL, Uniq, Inter	2041.505^***^ (1288)	0.861	0.845	0.064	0.058
M8 – FL, FVCV, Inter	2011.876^***^ (1276)	0.864	0.847	0.064	0.058
M9 – FL, Uniq, FVCV, Inter	2107.237^***^ (1303)	0.851	0.837	0.066	0.062
M10 – FL, Inter, Latent Means	1983.522^***^ (1266)	0.867	0.850	0.063	0.053
M11 – FL, Uniq, Inter, Latent Means	2049.126^***^ (1293)	0.860	0.845	0.064	0.058
M12 – FL, FVCV, Inter, Latent Means	2019.545^***^ (1281)	0.863	0.847	0.064	0.059
M13 – FL, Uniq, FVCV, Inter, Latent Means	2150.765^***^ (1308)	0.844	0.830	0.067	0.062

Unless indicated otherwise, *N* = 142. FL, Item factor loadings; Uniq, Item uniquenesses/residuals; Inter, Item intercepts; FVCV, Latent variable factor variance covariance matrix. ^***^p < 0.001.

#### Time invariance

The step 1 model, assessing configural invariance (i.e., whether the pattern of latent constructs was qualitatively invariant across the two times), showed a relatively poor fit. Constraining item factor loadings in the weak invariance model led to a more restrictive model and improved fit of indices susceptible to parsimony concerns (Morin et al., 2013), such as the TLI (∆TLI: 0.015) and the RMSEA (∆RMSEA: −0.003), but only increased the CFI by 0.001 and the SRMR by 0.010. As three out of four indicators were within bounds, we consider that weak invariance was confirmed. Comparing model 2 and model 5 showed little changes occurred in the indexes and supported the strong measurement invariance. For the strict measurement invariance models comparison, the change for the CFI went slightly over the proposed limit (∆CFI: - 0.006) but all other changes were within required limits. The model 2 and model 4 comparison supported factor variance–covariance invariance as all changes fell within chosen limits. All model pair comparisons assessing time mean invariance measurement showed changes below the cut-off scores, except for the comparison between model 9 and 13, with a ∆CFI of −0.007. However, it is noteworthy that models that constrained item uniqueness to be invariant systematically had a worse fit than those with other constraints, suggesting a difference in measurement error between Time 1 and Time 2. The constraints of covariance, latent means and item intercepts did not reduce model fit notably. As such, overall, the scale can be considered adequately time invariant across 6 weeks.

#### Test re-test reliability and validity

Table 11 shows the correlations for the MPSES subscales and the composite index between Time 1 and Time 2, as well as subscales intercorrelations and reliabilities for both times. Test–retest correlations for subscales range from 0.687 to 0.783, testifying to adequate test–retest reliability. Likewise, internal reliabilities varied from 0.826 to 0.892 for Time 1 and from 0.840 to 0.908 for Time 2, which is adequate. This table also shows the correlations between subscales at Time 1 and at Time 2. The scale showed, overall, a quasi-simplex structure at both times, as subscales adjacent to each other showed stronger correlations than more distal subscales, all in the expected directions. Finally, the correlation between the Time 1 and Time 2 index score was moderately strong. As our index score represents fluctuation in the saliency of different motivations and was assessed over a long period of time which included discrete achievement events such as exams, it was deemed acceptable.

**Table 11:** MPSES correlations and reliabilities, Study 2.

Subscale	α T1	α T2	ω T1	ω T2	Amotivation	P. External	P. Introjected	P. Identified	Excellence	Index
Amotivation	0.878	0.907	0.881	0.908	0.687^***^	0.290^***^	0.264^**^	0.073	−0.105	−0.737^***^
P. External	0.826	0.857	0.833	0.860	0.279^***^	0.738^***^	0.673^***^	0.374^***^	0.056	−0.355^***^
P. Introjected	0.876	0.891	0.877	0.893	0.279^***^	0.621^***^	0.783^***^	0.571^***^	0.210^*^	−0.131
P. Identified	0.887	0.840	0.892	0.851	−0.001	0.409^***^	0.592^***^	0.773^***^	0.736^***^	0.425^***^
Excellence	0.883	0.885	0.888	0.894	−0.090	0.166^**^	0.382^***^	0.779^***^	0.714^***^	0.708^***^
Index					−0.720^***^	−0.246^***^	0.010	0.537^***^	0.719^***^	0.441^***^

P., Perfectionism. Correlations between corresponding Time 1 (*N* = 287) and Time 2 (*N* = 142) subscales are on the diagonal (in bold). The correlations below the diagonal correspond to Time 1 data, and the correlations above the diagonal correspond to Time 2 data. T1 = Time 1; T2 = Time 2. ^*^p < 0.05, ^**^p < 0.01, ^***^p < 0.001.

Table 12 shows the correlations of the MPSES subscales and the composite index with the BFI-15 and the self-esteem item. The conscientiousness subscale was of particular interest, considering the results of Study 1b, in which a single item referring to striving for excellence strongly drove the correlation with introjected perfectionism. Conscientiousness correlated positively with identified perfectionism and with striving for perfection as well as negatively with amotivation. It also correlated positively with introjected perfectionism, albeit weakly. The correlation was driven by one item, which refers to “doing a thorough job.” As in Study 1b, neuroticism correlated positively with external perfectionism and introjected perfectionism, as well as with amotivation. The single item measure of self-esteem correlated negatively with external perfectionism and introjected perfectionism, and positively with identified perfectionism and striving for excellence. Finally, the index score also showed a positive relationship with conscientiousness and the self-esteem item, while correlating negatively with neuroticism.

**Table 12:** Correlations Between the MPSES and the BFI-15 and Self-Esteem Item, Study 2.

Subscale	O	C	E	A	N	SE
Amotivation	−0.075	−0.216^***^	0.024	−0.133^*^	0.256^***^	−0.251^***^
P. External	0.040	−0.001	−0.033	−0.033	0.363^***^	−0.269^***^
P. Introjected	0.006	0.131^*^	−0.047	−0.100	0.405^***^	−0.166^**^
P. Identified	0.041	0.526^***^	0.006	−0.092	0.089	0.173^**^
Excellence	0.077	0.530^***^	−0.001	−0.056	−0.032	0.222^***^
Index	0.084	0.532^***^	−0.005	0.037	−0.245^***^	0.378^***^

P., Perfectionism; O, Openness; C, Conscientiousness; E, Extraversion; A, Agreeableness; N, Neuroticism; SE, Self-esteem. ^*^p < 0.05, ^**^p < 0.01, and ^***^p < 0.001.

A *t*-test showed no difference on the index score between people who answered at both times and only at Time 1, *t*(285) = −1.623, *p* = 0.106. In line with the analyses for Study 1a, we conducted a GLM analysis with age and gender as predictors on the full sample (see Supplementary material for details). Overall, results showed an effect of age, qualified by an interaction with gender, such as the MPSES score increased more for men than women. Subscale level analyses also showed a decrease in amotivation and external perfectionism as age increased, and differing levels of increase in striving for excellence by gender.

## General discussion

The present set of studies aimed to create a new framework for assessing perfectionism as a motivational process. Our perspective combined the SDT view with personality development theories relating to self-definition, as suggested by Blatt and colleagues (Shahar et al., 2003; Luyten and Blatt, 2011, 2016). Based on this, the MPSES includes constructs such as amotivation, representing a lack of meaningful striving, and striving for excellence, underlain by intrinsic motivation. In between these extremes, we posited the existence of three forms of extrinsically regulated perfectionism: external perfectionism, introjected perfectionism, and identified/integrated perfectionism. Introjected perfectionism was thought to be the common variance between PC, underlain by external perfectionism, and PS, underlain by identified/integrated perfectionism. Following the creation of the new questionnaire, we assessed model fit using ESEM. The resulting model showed acceptable properties. The model was retested using a shortened scale in a new sample of students and showed once more an acceptable fit. The scale was also shown to be time invariant and to have good test–retest reliability. In both studies, the scale followed a quasi-simplex structure and showed acceptable reliabilities. Finally, examination of correlational profiles with related constructs overall fell within expected parameters, both at the subscale level and for the full scale.

Results of the convergent and divergent validity analyses supported the underlying continuum of internalization and of self-definition that we proposed. Only identified perfectionism and striving for excellence were associated with an increased feeling of satisfaction about oneself and were either associated with a decreased level of self-derogation or not associated with self-derogation at all. Both findings are suggestive of a better self-definition. As such, the higher neuroticism and self-derogation, as well as lessened satisfaction about oneself associated with external and introjected perfectionism imply self-definition issues.

Conversely, our results also showed introjected perfectionism to be associated with the conscientiousness subscale of the NEO-FFI and of the BFI-15. Further examination of items driving these correlations referred to performing or working hard. It is not incoherent that individuals driven mainly by ego related motives would also wish to achieve excellence or get the job done. Hence, introjected perfectionism fell in the in-between zone suggested by other researchers (Howard et al., 2017; Vasconcellos et al., 2019). It correlated with both positively valenced and negatively valenced variables, and with both internal and external standards, such as the SOP and SPP subscales, or the importance of reaching one’s own and others’ goals and standards. These results further lent credence to the view that PS is characterized by some level of ego involvement. Research has shown PS to be associated with more self-determined forms of extrinsic motivation, as well as with intrinsic motivation (Miquelon et al., 2005; Stoeber et al., 2009, 2013; Chang et al., 2016; Hill et al., 2018). However, it is also associated with more controlled forms of motivation, such as introjected motivation (Longbottom et al., 2012; Stoeber et al., 2013; Hill et al., 2018), and has been associated with external rewards, competition or recognition (Mills and Blankstein, 2000).

These results further align with the association of both introjected perfectionism and identified perfectionism with the control orientation (i.e., pressure) subscale of the GOCS. In the academic context, a feeling of pressure can stem from a desire for advancement in one’s field of study, or to stand apart from peers in the hope of reaching higher education; or to become the best researcher or clinician one can hope to be. As identified perfectionism depicts a level of internalization in which the standard is important and coherent with the person’s goals or values, while still being instrumental in some way, a sense of pressure remains. Accordingly, it has been suggested that PS behaviors result from a form of internalized pressure to attain high standards, and not only felt interest or self-determined choice (Flett and Hewitt, 2006). In opposition, the striving for excellence subscale, which correlated with the controlled orientation, also correlated with the autonomy orientation, suggesting the presence of a level of choice in seeking the highest degree of performance. This differs from the external and introjected perfectionism subscales, which correlated with the impersonal orientation of the GCOS, characterized by a perceived lack of control over desired outcomes, due for example to a lack of competence (Deci and Ryan, 1985a). It could therefore be that striving for excellence, and to some degree identified perfectionism, are underlain by some perceived competence in reaching a desired level of performance, and that these experiences of success contributed to the internalization process of the standard.

Wishing to do well and wanting to reach one’s own or others’ goals and standards were overall common to all forms of striving, and even, somewhat surprisingly, to amotivation. Amotivation’s association with other’s goals and standards suggests some reliance on external standards for guidance. However, only identified perfectionism and striving for excellence was characterized by goal setting and behaviors conductive to reaching these goals. Associations with the MAA scale revealed that individuals with introjected perfectionism aimed to have high standards without actively pursuing this goal. Likewise, external perfectionism was only associated with thinking about one’s values when facing goal conflicts, again without active pursuit. The degree to which actual behavior conductive to striving is shown, versus simply setting high standards or wanting to reach these standards, could therefore also be a distinctive characteristic that distinguishes between dimensions of perfectionism and striving for excellence.

Furthermore, whereas both identified perfectionism and striving for excellence were associated with more active engagement on the MAA scale, they also differed in important ways. Further examination of the strength of the correlations with different subscales suggested that identified perfectionists may try harder, for example, by setting high standards in more classes and making more specific efforts, whereas individuals striving for excellence may be more driven by interest and a desire to learn, all the while enjoying intrinsic rewards in the process of their striving.

Following this rationale, it is coherent that introjected perfectionism was associated with an avoidance goal in the form of the lowest standard of performance, as assessed in Study 1b. This form of perfectionism is anchored in avoiding negative outcomes so as to avoid negative contingencies. The association of this goal with identified perfectionism then suggests that failure does remain a concern with this dimension of perfectionism, but that it occurs alongside an approach goal, or a hope for success. These results connect with past research showing PS to be related to performance approach goals (Eum and Rice, 2011; Hill et al., 2018), and inconsistently with avoidance goals (Verner-Filion and Gaudreau, 2010; Speirs Neumeister et al., 2015), as well as with hope for success (Stoeber and Rambow, 2007; Stoeber and Becker, 2008), reactivity when facing failure (Flett et al., 2016) and low tolerance for failure and disapproval (Flett et al., 1991). Nonetheless, as others’ lowest and ideal standards of performance did not correlate at all with the scale, these results suggest that perfectionism, no matter its motivational components, is driven by some level of endorsement of the goals. Consequently, this also supports the notion that the external subscale of the MPSES does not only assess individuals’ perception that others’ hold high standards for them, but their actual endorsement of this goal, as weak as it might be.

Results for the GLM analyses of gender and age on the MPSES were mixed. Models were only significant for Study 2 and showed that, overall, individuals become more self-determined in their striving as they age. They further indicated that men experience a larger increase in MPSES score and in striving for excellence than women. However, these effects could be the result of a self-selection bias in our sample. Individuals whose scholastic perfectionism profile is more self-determined may be more likely to further their education as they age.

The studies presented were somewhat limited in scope as a result of sample size issues. In particular, the sample aiming to assess the nomological net of the scale was smaller than ideal. Thus, even though the significant results were aligned with predictions, we were not able to reliably detect smaller effects. Likewise, the model we proposed was complex for the size of the sample it was assessed with.

Future research will thus be instrumental in producing further support for the fit of our model and the usefulness of the scale, including research designs measuring the predictive ability of the questionnaire. Our results also suggest that more research is needed in assessing psychological functioning and behavioral components which defines and differentiate dimensions of perfectionism. Specifically, we found evidence that, while perfectionists indicate they hold these standards, they might not all strive actively to reach them.

## Conclusion

In sum, the model underlying the MPSES showed proper fit with two samples. The scale showed a quasi-simplex structure suggestive of a continuum. It also showed good reliabilities and was found to be time invariant. Finally, it showed good convergent and divergent validity with selected constructs. These results provided support for the structure of the questionnaire. Therefore, the MPSES is a new and promising scale for the study of perfectionism from a motivational perspective. Indeed, it explicitly measures perfectionism as a motive, in comparison with other scales where the measurement of motivational components is more implicit. The framework also proposes a solution as to the shared component between PS and PC, in the form of an underlying continuum of self-definition. Broadly, this adds to past research implying that perfectionism, itself, might not be the disease, but rather the manifestation of an adaptation in a core human structure – identity and self-esteem.

## Transparency and openness

We report how we determined our sample sizes, all data exclusions, all manipulations, and all measures in the study, and we follow JARS (Kazak, 2018). An example of ESEM analysis code and an example of GLM assumptions code are available in the Supplementary material, as are the MPSES items. The ESEM model and invariance models were analyzed using Mplus 8.0 (Muthén and Muthén, 2017) whereas all other analyses were run using the program jamovi 1.1.5 (The jamovi project, 2019). However, assumptions for GLM analyses were checked using RStudio (RStudio Team, 2020) with the ggResidpanel (Goode and Rey, 2019), the performance (Lüdecke et al., 2021), and the stats (R Core Team, 2021) package. Cook’s distance and leverage were assessed using a R syntax by Silk (2019), which is freely available. These studies were not preregistered.

## Data availability statement

The raw data supporting the conclusions of this article will be made available by the authors, without undue reservation.

## Ethics statement

The studies involving human participants were reviewed and approved by University of Quebec in Montreal Departmental Ethics Committee, University of Quebec in Montreal Research Ethics Committee for Student Projects (CERPE 4—UQAM), and the University of Quebec in Abitibi-Témiscamingue Research Ethics Committee (CÉR-UQAT). The patients/participants provided their written informed consent to participate in this study.

## Author contributions

ML and UH contributed to the conception, design of the studies, and performed statistical analyses. ML collected the data and organized the databases. ML wrote the first draft of the manuscript. All authors contributed to the article and approved the submitted version.

## Funding

This research was supported in part by a graduate student scholarship from the Fonds de recherche du Québec—Société et culture (FQRSC).

## Conflict of interest

The authors declare that the research was conducted in the absence of any commercial or financial relationships that could be construed as a potential conflict of interest.

## Publisher’s note

All claims expressed in this article are solely those of the authors and do not necessarily represent those of their affiliated organizations, or those of the publisher, the editors and the reviewers. Any product that may be evaluated in this article, or claim that may be made by its manufacturer, is not guaranteed or endorsed by the publisher.
