# Supplementary material for: A motivational approach to perfectionism and striving for excellence: Development of a new continuum-based scale for post-secondary students
Source: Front Psychol. 2022 Nov 10;13:1022462. doi: 10.3389/fpsyg.2022.1022462 (PMC9686388; doi:10.3389/fpsyg.2022.1022462)
Supplement: Supplementary file 1 [file Table_1.docx]

Supplementary Material

The supplementary material first provides a full list of all Multidimensional Perfectionism and Striving For Excellence Scale (MPSES) items in French and in English. It is followed by an example of the Mplus code used to analyze the ESEM models, and an example of the RStudio code used to assess GLM assumptions. Model assumptions were verified using RStudio (RStudio Team, 2020) with the ggResidpanel (Goode & Rey, 2019), the performance (Lüdecke et al., 2021) and the stats (R Core Team, 2021) package. Cook’s distance and leverage were assessed using a R syntax by Silk (2019), which is freely available online. Table S1 provides correlated errors for Study 1a. Table S2 provides items loadings for Study 1a. Table S3 provides reliabilities for the scales used to assess validity in Study 1b and Study 2. Table S4 provides the partial correlations between the MPSES and self-oriented perfectionism and socially prescribed perfectionism. The last section details the GLM analyses of age and gender on the MPSES for Study 1a and Study 2. Table S5 and Table S6 show the results of the significant models for Study 2. Figure S1 to S15 show the results of assumption checks for all GLM analyses (Study 1a and Study 2).

*Questionnaire Items (in French)*

**Questionnaire de standards élevés**

Voici une liste d’affirmations qui se rapportent à vos standards dans la poursuite de vos études. Veuillez encercler le chiffre qui correspond le mieux à votre degré d'accord ou de désaccord avec chacune de ces affirmations. Utilisez l'échelle d'évaluation suivante:

| **1** | **2** | **3** | **4** | **5** | **6** | **7** |
| --- | --- | --- | --- | --- | --- | --- |
| **Totalement en désaccord** | **Fortement en**  **Désaccord** | **Plutôt en**  **désaccord** | **Indécis** | **Plutôt en**  **accord** | **Fortement en**  **accord** | **Totalement en accord** |

| 1. | Je ne crois pas avoir d’autre choix que de m'efforcer d'atteindre les attentes élevées des autres. | 1 2 3 4 5 6 7 |
| --- | --- | --- |
| 2. | Je dois m’efforcer d’atteindre l’excellence afin de m’assurer d’obtenir de bonnes notes. | 1 2 3 4 5 6 7 |
| 3. | Faire des erreurs dans mes examens ou travaux me fait ressentir de la culpabilité. | 1 2 3 4 5 6 7 |
| 4. | Je n’établie pas de buts élevés pour moi-même. | 1 2 3 4 5 6 7 |
| 5. | Je ne demande rien de moins que l’excellence de moi-même car je suis un bon étudiant, et c’est ce qu’un bon étudiant fait. | 1 2 3 4 5 6 7 |
| 6. | Je me sentirai très mal face à moi-même si je ne remets pas un travail de la plus haute qualité. | 1 2 3 4 5 6 7 |
| 7. | Je ressens de la pression à faire mes travaux scolaires parfaitement. | 1 2 3 4 5 6 7 |
| 8. | J’essaie d’atteindre mon plein potentiel dans mes travaux scolaires car cela constitue un défi personnel pour moi. | 1 2 3 4 5 6 7 |
| 9. | Je n’exige pas l’excellence de moi-même. | 1 2 3 4 5 6 7 |
| 10. | Je crois fermement que n’importe quoi en deçà de l’excellence n’est pas acceptable. | 1 2 3 4 5 6 7 |
| 11. | Je me fixe les plus hauts standards car j’aime me lancer des défis. | 1 2 3 4 5 6 7 |
| 12. | J’aurai honte de moi si je ne travaille pas à mon plein potentiel. | 1 2 3 4 5 6 7 |
| 13. | J’essaie de faire mon travail scolaire parfaitement car j’aime tester mes limites. | 1 2 3 4 5 6 7 |
| 14. | Ne pas m’efforcer d’être à mon meilleur irait contre qui je suis. | 1 2 3 4 5 6 7 |
| 15. | Je donne mon meilleur car j’aime être à mon meilleur. | 1 2 3 4 5 6 7 |
| 16. | Je suis un bon étudiant, et être un bon étudiant signifie que je dois toujours produire un travail de qualité supérieure. | 1 2 3 4 5 6 7 |
| 17. | Je ressens de la fierté si je fais un projet ou examen parfaitement. | 1 2 3 4 5 6 7 |
| 18. | Je travaille à mon plein potentiel à l’école car l’éducation est l’une de mes valeurs. | 1 2 3 4 5 6 7 |
| 19. | J’essaie d’exceller car je veux que mes professeurs aient une bonne opinion de moi. | 1 2 3 4 5 6 7 |
| 20. | Faire les choses de façon moins qu’impeccable me fait sentir coupable. | 1 2 3 4 5 6 7 |
| 21. | Les autres m’aimeront davantage si j’ai du succès à l’école. | 1 2 3 4 5 6 7 |
| 22. | J’essaie de faire mon travail scolaire parfaitement car j’aime repousser mes limites. | 1 2 3 4 5 6 7 |
| 23. | Je fais tous les efforts afin d’atteindre l’excellence, car cela fait partie de qui je suis. | 1 2 3 4 5 6 7 |
| 24. | Je dois atteindre les standards élevés des autres sinon j’aurai des problèmes. | 1 2 3 4 5 6 7 |
| 25. | Je n’ai pas de standards élevés pour mes travaux scolaires. | 1 2 3 4 5 6 7 |
| 26. | Je ne donne rien sauf mon meilleur à l’école car c’est ce que je suis supposé faire. | 1 2 3 4 5 6 7 |
| 27. | Je dois exceller dans mes travaux scolaires car je ne veux pas que les autres soient déçus de moi. | 1 2 3 4 5 6 7 |
| 28. | Je vais me détester si je n’atteins pas mes attentes élevées. | 1 2 3 4 5 6 7 |
| 29. | Je m’efforce de produire une performance exceptionnelle à cause du défi que cela m’apporte. | 1 2 3 4 5 6 7 |
| 30. | Je crois personnellement qu’il est important pour moi de me fixer les standards les plus élevés. | 1 2 3 4 5 6 7 |
| 31. | Je ne suis pas certain pourquoi je m’efforce d’être le meilleur. | 1 2 3 4 5 6 7 |
| 32. | J’essaie d’exceller à l’école car je désire apprendre. | 1 2 3 4 5 6 7 |
| 33. | Je ne demande de moi-même que le meilleur car autrement je me sentirais mal dans ma peau. | 1 2 3 4 5 6 7 |
| 34. | Il est important pour moi de donner mon meilleur à l’école. | 1 2 3 4 5 6 7 |
| 35. | Je m’efforce d’avoir du succès à l’école car il est important pour moi de bien faire. | 1 2 3 4 5 6 7 |
| 36. | Il n’est pas clair pour moi pourquoi j’essaie d’avoir du succès dans ce que je fais. | 1 2 3 4 5 6 7 |
| 37. | Je suis exigent envers moi-même dans l’établissement de mes buts car cela fait partie de qui je suis. | 1 2 3 4 5 6 7 |
| 38. | Je ne sais pas pourquoi je ressens le besoin d’exceller. | 1 2 3 4 5 6 7 |
| 39. | Je dois exceller dans tout ce que je fais à l’école afin d’atteindre mes buts futurs. | 1 2 3 4 5 6 7 |
| 40. | Je ne me sentirai pas bien face à moi-même sauf si ma performance est supérieure à la moyenne. | 1 2 3 4 5 6 7 |
| 41. | Je dois m’efforcer d’atteindre l’excellence afin d’éviter d’avoir de mauvaises notes. | 1 2 3 4 5 6 7 |
| 42. | Produire un travail scolaire qui n’atteint pas l’excellence me fait sentir comme un échec. | 1 2 3 4 5 6 7 |
| 43. | Je veux atteindre des buts extrêmement élevés car cela fait partie de moi. | 1 2 3 4 5 6 7 |

*Questionnaire Items (in English)*

Listed below are statements about your standards in the pursuit of your studies. Please click on the number best representing your degree of agreement or disagreement with each statement based on the provided scale.

| **1** | **2** | **3** | **4** | **5** | **6** | **7** |
| --- | --- | --- | --- | --- | --- | --- |
| **Totally Disagree** | **Strongly Disagree** | **Somewhat Disagree** | **Neither Agree nor Disagree** | **Somewhat Agee** | **Strongly**  **Agree** | **Totally**  **Agree** |

| 1. | I don’t feel I have a choice but to strive to attain other’s high expectations. | 1 2 3 4 5 6 7 |
| --- | --- | --- |
| 2. | I have to strive for excellence to make sure to get good grades. | 1 2 3 4 5 6 7 |
| 3. | Doing mistakes in my exams or school work makes me feel guilty. | 1 2 3 4 5 6 7 |
| 4. | I don’t set the highest goals for myself. | 1 2 3 4 5 6 7 |
| 5. | I demand nothing short of excellence for myself because I’m a good student, and that’s what a good student does. | 1 2 3 4 5 6 7 |
| 6. | I’ll feel very bad about myself if I don’t give in top notch quality work. | 1 2 3 4 5 6 7 |
| 7. | I feel pressured to do my school work flawlessly. | 1 2 3 4 5 6 7 |
| 8. | I try to reach my full potential in my schoolwork because it’s a personal challenge. | 1 2 3 4 5 6 7 |
| 9. | I don’t demand excellence of myself. | 1 2 3 4 5 6 7 |
| 10. | I have a strong value that anything less than excellence is not acceptable. | 1 2 3 4 5 6 7 |
| 11. | I set the highest standards because I enjoy challenging myself. | 1 2 3 4 5 6 7 |
| 12. | I’ll be ashamed of myself if I don’t work at my full potential. | 1 2 3 4 5 6 7 |
| 13. | I try to do my work flawlessly because I enjoy testing my limits. | 1 2 3 4 5 6 7 |
| 14. | Not striving to be my best would go against who I am. | 1 2 3 4 5 6 7 |
| 15. | I do my absolute best because I enjoy being my best. | 1 2 3 4 5 6 7 |
| 16. | I’m a good student, and being a good student means I must always produce top-notch quality work. | 1 2 3 4 5 6 7 |
| 17. | I feel proud if I do a school project or an exam flawlessly. | 1 2 3 4 5 6 7 |
| 18. | I work at my full potential at school because I value education. | 1 2 3 4 5 6 7 |
| 19. | I try to excel because I want my professors to think well of me. | 1 2 3 4 5 6 7 |
| 20. | Doing things less than impeccably makes me feel guilty. | 1 2 3 4 5 6 7 |
| 21. | People will like me better if I’m successful at school. | 1 2 3 4 5 6 7 |
| 22. | I try to do my work flawlessly because I enjoy pushing my limits. | 1 2 3 4 5 6 7 |
| 23. | I try my hardest to reach excellence because it’s part of who I am. | 1 2 3 4 5 6 7 |
| 24. | I have to meet other’s high standards for me, or I’ll get in troubles. | 1 2 3 4 5 6 7 |
| 25. | I don’t have high standards for my schoolwork. | 1 2 3 4 5 6 7 |
| 26. | I do nothing but my best at school because it’s what I’m supposed to do. | 1 2 3 4 5 6 7 |
| 27. | I have to excel at school work because I don’t want people to be disappointed in me. | 1 2 3 4 5 6 7 |
| 28. | I’ll hate myself if I don’t reach my high expectations. | 1 2 3 4 5 6 7 |
| 29. | I strive to do an outstanding performance because of the challenge. | 1 2 3 4 5 6 7 |
| 30. | I personally believe it’s important to set the highest standard for myself. | 1 2 3 4 5 6 7 |
| 31. | I don’t know why I feel the need to excel. | 1 2 3 4 5 6 7 |
| 32. | I try to excel at school because I want to learn. | 1 2 3 4 5 6 7 |
| 33. | I demand nothing but the best of myself because otherwise I would feel bad about myself. | 1 2 3 4 5 6 7 |
| 34. | It’s important to me to do my absolute best at school. | 1 2 3 4 5 6 7 |
| 35. | I strive to be successful at school because it’s important for me to do well. | 1 2 3 4 5 6 7 |
| 36. | It’s not clear why I try to succeed at what I do. | 1 2 3 4 5 6 7 |
| 37. | I am demanding of myself in setting my goals because it is who I am. | 1 2 3 4 5 6 7 |
| 38. | I am not sure why I strive to be the best. | 1 2 3 4 5 6 7 |
| 39. | I must excel in everything I do at school in order to reach my future endeavours. | 1 2 3 4 5 6 7 |
| 40. | I won’t feel good about myself unless I do much better than an average performance. | 1 2 3 4 5 6 7 |
| 41. | I have to strive for excellence to avoid getting bad grades. | 1 2 3 4 5 6 7 |
| 42. | Doing any school work short of excellence make me feel like a failure. | 1 2 3 4 5 6 7 |
| 43. | I want to reach extremely high goals because it’s part of who I am. | 1 2 3 4 5 6 7 |

*Example of ESEM Syntax, Study 1a*

TITLE: ESEM Target Rotation

DATA: FILE = basemodel.txt;

VARIABLE:

NAMES = ID AMO1-AMO6 EXT1-EXT9 Ident1-Ident7 Integ1-Integ6

INTRIN1-INTRIN6 Introj1-Introj9;

USEVAR = AMO1-AMO6 EXT1-EXT9 Ident1-Ident7 Integ1-Integ6

INTRIN1-INTRIN6 Introj1-Introj9;

Missing AMO1-Introj9 (-9999);

Analysis: ESTIMATOR = MLR; ROTATION=TARGET;

Model:

Amotiv by AMO1-AMO6 EXT1-EXT9~0 Introj1-Introj9~0 Ident1-Ident7~0

Integ1-Integ6~0 INTRIN1-INTRIN6~0 (*1);

Ext by EXT1-EXT9 AMO1-AMO6~0 Introj1-Introj9~0 Ident1-Ident7~0

Integ1-Integ6~0 INTRIN1-INTRIN6~0 (*1);

Introj by Introj1-Introj9 AMO1-AMO6~0 EXT1-EXT9~0 Ident1-Ident7~0

Integ1-Integ6~0 INTRIN1-INTRIN6~0 (*1);

Ident by Ident1-Ident7 Integ1-Integ6 AMO1-AMO6~0 EXT1-EXT9~0 Introj1-Introj9~0

INTRIN1-INTRIN6~0 (*1);

Intrin by INTRIN1-INTRIN6 AMO1-AMO6~0 EXT1-EXT9~0 Introj1-Introj9~0

Ident1-Ident7~0 Integ1-Integ6~0 (*1);

Output: sampstat standardized SVALUES stdyx TECH2 tech4 mod res;

*Example of Syntax For GLM Assumptions in RStudio*

*#Syntax to obtain data from gamlj model*

MPSES267 <- read_csv("MPSES267.csv", col_names = TRUE,

trim_ws = FALSE)

gmod1<- gamljGLM(formula = MPSES_tot ~ genre + age + genre:age, data = MPSES267)

gdata1<-gamlj_data(gmod1)

write.csv(gdata1, file = "MPSES267_M.csv")

#Preparation#

f.gender <- factor(MPSES267_M$gender)

s.age <- (MPSES267_M$age)

contrasts(f.genre)<-contr.treatment(2)-1/2

#Model#

mod_MPSES <- lm(MPSES_tot ~ 1 + f.gender + s.age + f.gender:s.age, data = MPSES267_M)

summary(mod_MPSES)

car::Anova(mod_MPSES, type = "III")

#Assumptions#

library(performance)

check_model(mod_MPSES, check = "linearity")

check_model(mod_MPSES, check = "homogeneity")

check_model(mod_MPSES, check = "vif")

check_model(mod_MPSES, check = "normality")

check_model(mod_MPSES, check = "qq")

library(ggResidpanel)

resid_xpanel(mod_MPSES)

#Outliers (Silk, 2019)

#Leverage

lev<-hat(model.matrix(mod_MPSES))

#Cook's Distance

cd<-cooks.distance(mod_MPSES)

#Plot leverage and Cook's distance together

par(mfrow=c(1,1))

plot(lev,pch=16,col="red",ylim=c(0,1.2),las=1,ylab="Leverage/Cook's distance value")

points(cd,pch=17,col="blue")

points(x=150,y=1.1,pch=16,col="red")

points(x=150,y=0.9,pch=17,col="blue")

text(x=155,y=1.1,"Leverage",adj=c(0,0.5))

text(x=155,y=0.9,"Cook's distance",adj=c(0,0.5))

title(main = "Outlier Detection", cex.main = 1)

**Table S1**

Correlated Errors, Study 1a

| Item # 1 |  | Item # 2 |  |
| --- | --- | --- | --- |
| Intro20 | Doing things less than impeccably makes me feel guilty. | Intro3 | Doing mistakes in my exams or school work makes me feel guilty. |
| Intro28 | I’ll hate myself if I don’t reach my high expectations. | Intro33 | I demand nothing but the best of myself because otherwise I would feel bad about myself. |
| Integ 14 | Not striving to be my best would go against who I am. | Integ5 | I demand nothing short of excellence for myself because I’m a good student, and that’s what a good student does. |
| Integ23 | I try my hardest to reach excellence because it’s part of who I am. | Integ37 | I am demanding of myself in setting my goals because it is who I am. |

**Table S2**

Items Loadings (and Removed Items) Study 1a

| Item | Amotivation | P. External | P. Introjected | P. Identified | Excellence | Removal |
| --- | --- | --- | --- | --- | --- | --- |
| Amo25 | 0.101 | **0.227** | **-0.374** | **-0.634** | -0.098 | Removed |
| Amo31 | **1.251** | -0.091 | 0.106 | 0.009 | -0.015 |  |
| Amo36 | **1.337** | 0.113 | -0.044 | -0.117 | 0.130 |  |
| Amo38 | **1.305** | -0.095 | 0.096 | -0.054 | 0.054 |  |
| Amo4 | 0.085 | 0.264 | -0.130 | **-0.339** | **-0.315** | Removed |
| Amo9 | 0.085 | 0.048 | **-0.914** | -0.213 | -0.224 | Removed |
| Ext1 | 0.137 | **0.717** | 0.033 | 0.267 | -0.209 |  |
| Ext19 | -0.061 | **0.906** | -0.126 | **0.330** | 0.170 |  |
| Ext2 | 0.113 | **0.275** | 0.211 | **0.563** | 0.076 | Removed |
| Ext21 | **-0.232** | **1.267** | 0.234 | **-0.439** | 0.112 |  |
| Ext24 | 0.039 | **1.164** | 0.177 | **-0.239** | -0.002 |  |
| Ext26 | -0.020 | **0.454** | 0.200 | **0.627** | 0.064 | Removed |
| Ext27 | **0.279** | **1.520** | -0.236 | 0.055 | 0.021 |  |
| Ext41 | 0.071 | **0.409** | **0.552** | **0.631** | -0.117 | Removed |
| Ext7 | 0.192 | 0.201 | **0.851** | 0.247 | 0.089 | Removed |
| Intro12 | **0.220** | 0.184 | **0.757** | 0.272 | 0.038 |  |
| Intro17 | -0.023 | -0.017 | 0.018 | **0.375** | 0.008 | Removed |
| Intro20 | 0.037 | **0.384** | **0.865** | 0.200 | 0.070 |  |
| Intro28 | 0.101 | **0.690** | **0.999** | -0.225 | 0.038 |  |
| Intro3 | 0.158 | **0.461** | **0.719** | 0.137 | -0.121 |  |
| Intro33 | 0.122 | **0.421** | **0.803** | 0.348 | 0.161 |  |
| Intro40 | 0.123 | **0.288** | **0.979** | 0.233 | -0.179 |  |
| Intro42 | 0.033 | **0.396** | **1.213** | 0.052 | 0.087 |  |
| Intro6 | 0.059 | -0.101 | **0.826** | 0.296 | 0.128 |  |
| Ident10 | -0.096 | 0.283 | **0.905** | -0.164 | 0.224 | Removed |
| Ident18 | 0.024 | 0.095 | **-0.350** | **0.702** | **0.341** |  |
| Ident30 | -0.039 | 0.031 | 0.184 | **0.391** | **0.892** | Removed |
| Ident32 | **-0.152** | 0.071 | **-0.312** | **0.671** | 0.135 |  |
| Ident34 | -0.053 | 0.120 | 0.031 | **0.928** | 0.006 |  |
| Ident35 | **-0.169** | **0.187** | 0.091 | **0.892** | -0.127 |  |
| Ident39 | -0.024 | **0.422** | 0.230 | **0.794** | 0.086 |  |
| Integ14 | -0.085 | 0.125 | **0.458** | **0.467** | **0.442** |  |
| Integ16 | -0.033 | 0.203 | **0.399** | **0.620** | 0.106 |  |
| Integ23 | 0.029 | -0.085 | **0.566** | **0.600** | **0.584** |  |
| Integ37 | **-0.226** | -0.125 | 0.391 | **0.513** | **0.404** |  |
| Integ43 | -0.114 | -0.086 | **0.597** | **0.417** | **0.823** | Removed |
| Integ5 | 0.082 | **0.302** | **0.461** | **0.567** | 0.173 |  |
| Intrin11 | **-0.197** | -0.129 | -0.004 | 0.118 | **1.016** |  |
| Intrin13 | 0.018 | 0.070 | -0.019 | 0.076 | **1.250** |  |
| Intrin15 | -0.011 | 0.027 | 0.052 | **0.583** | **0.345** | Removed |
| Intrin22 | -0.034 | 0.097 | **-0.165** | 0.039 | **1.310** |  |
| Intrin29 | -0.052 | 0.171 | -0.166 | 0.012 | **1.242** |  |
| Intrin8 | -0.061 | -0.069 | -0.139 | **0.743** | **0.424** |  |

*Note.* P. = Perfectionism. Significant weights are in bold. Grey cells indicate items’ corresponding factor.

**Table S3**

Reliabilities for Scales Used to Assess Convergent and Divergent Validity (Study 1B and Study 2)

|  | Study 1B | | Study 2 | | |  |
| --- | --- | --- | --- | --- | --- | --- |
| Subscale | α | ω | | α | ω | |
| Openness | .711 | .710 | | .803 | .828 | |
| Conscientiousness | .833 | .852 | | .581 | .607 | |
| Extraversion | .777 | .798 | | .817 | .820 | |
| Agreeableness | .740 | .749 | | .500 | .573 | |
| Neuroticism | .842 | .848 | | .740 | .759 | |
| Self-Derogation | .830 | .844 | |  |  | |
| Social Desirability | .582 | .574 | |  |  | |
| Self-Oriented Perfectionism | .922 | .923 | |  |  | |
| Socially Prescribed Perfectionism | .906 | .915 | |  |  | |
| Other-Oriented Perfectionism | .829 | .839 | |  |  | |
| Autonomy Orientation | .858 | .869 | |  |  | |
| Control Orientation | .581 | .610 | |  |  | |
| Impersonal Orientation | .774 | .784 | |  |  | |

**Table S4**

Partial Correlations for SOP and SPP with the MPSES (Study 1B)

| Subscale | SOP | SPP |
| --- | --- | --- |
| Amotivation | -.192 | .270** |
| P. External | .221* | .607*** |
| P. Introjected | .672*** | .262** |
| P. Identified | .616*** | -.195 |
| Excellence | .398*** | -.241* |
| Index | .385*** | -.439*** |

Note. P. = Perfectionism.

*p < .05, **p < .01, ***p < .001

**Analyses**

*GLM of MPSES with Gender by Age (Study 1a)*

We assessed the effect of gender and age on the MPSES index score. A full factorial model was analyzed. The model was not significant, *F*(3, 262) = 2.289, *p* = .079, *η^2^p* = .026. Graphs (Figure S1 to S3) show the model overall respected all assumptions. While the model showed some linearity of residuals departure, this was expected as one of our predictor is binary (i.e., gender) and the other has a limited range of values (i.e., age).

**Figure S1**

*
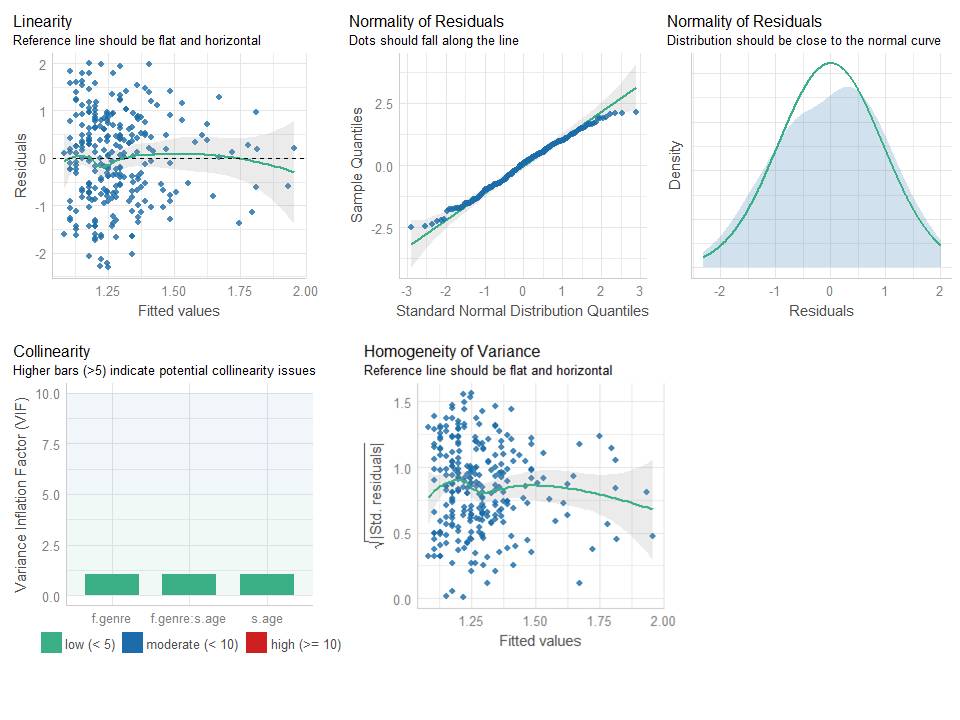
Collinearity and Homogeneity, Linearity, and Normality of Residuals of the MPSES by Age and Gender for Study 1a*

***Figure S2***

*Leverage and Cook’s Distance of the MPSES by Age and Gender for Study 1a*

*
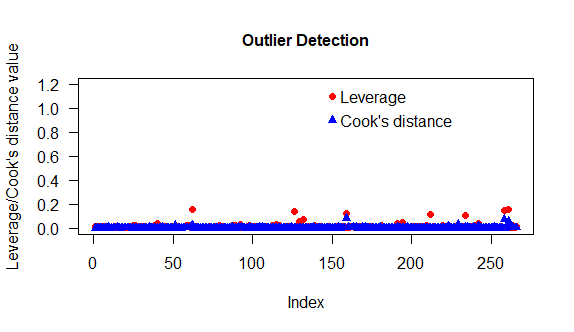
*

***Figure S3***

*Residuals per Predictor of the MPSES by Age and Gender for Study 1a*

**
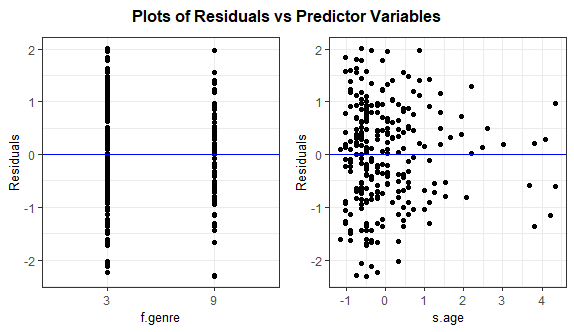
**

*GLM of MPSES by Gender and Age (Study 2)*

We assessed the effect of gender and age on the MPSES score (Table S5). A full factorial model was analyzed. The model was significant, *F*(3, 282) = 9.268, *p* < .001, *η^2^p* = .090. Results showed a significant main effect of age, *F*(1, 282) = 23.222, *p* < .001, *η^2^p* = .072, which was qualified by an interaction with gender, *F*(1, 282) = 3.991, *p* = .047, *η^2^p* = .014. Simple effect analyses showed that an increase in age was associated with a bigger increase on the index scale for men, *F*(1, 282) = 14.017, *p* < .001, *η^2^p* = .047, than for women, *F*(1, 282) = 11.620, *p* < .001, *η^2^p* = .040. Graphs (Figure S4 to S6) showed the model overall respected all assumptions.

**Table S5**

GLM of MPSES by Gender and Age (Study 2)

|  | Estimate | SE | 95% IC | | *P* | | Β | |  |
| --- | --- | --- | --- | --- | --- | --- | --- | --- | --- |
|  |  |  | LL | UL |  | |  |  |  |
| Gender | .207 | .148 | -.085 | .498 | .163 | .210 | | | |
| Age | .040 | .008 | .024 | .056 | <.001 | .363 | | | |
| Gender x Age | .033 | .017 | 4.847e-4 | .066 | .047 | .301 | | | |
| Women | .023 | .007 | .010 | .037 | <.001 |  | | | |
| Men | .057 | .015 | .027 | .086 | <.001 |  | | | |

|  |  |
| --- | --- |
|  | |

**Figure S4**

*
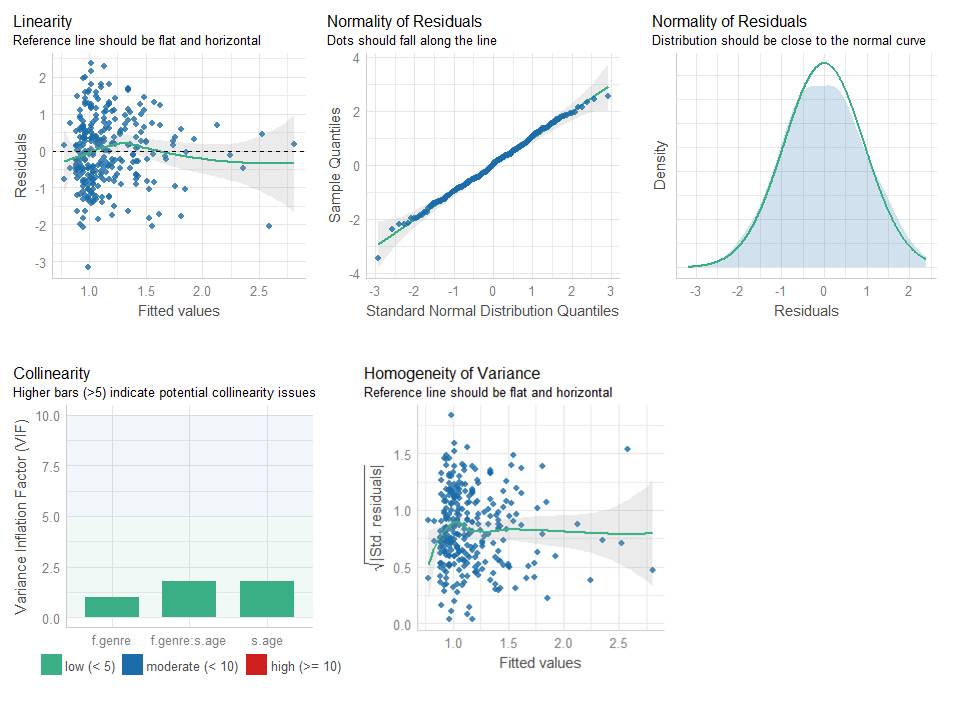
Collinearity and Homogeneity, Linearity and Normality of Residuals of the MPSES by Age and Gender for Study 2*

***Figure S5***

*Leverage and Cook’s Distance of the MPSES by Age and Gender for Study 2*

**
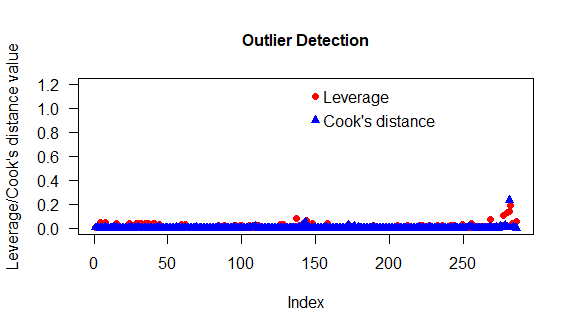
**

***Figure S6***

*Residuals per Predictor of the MPSES by Age and Gender for Study 2*

**
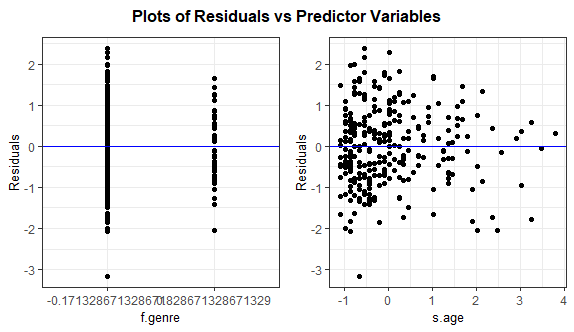
**

  Conducting this analysis for each subscale (Table S6) showed that only the amotivation, *F*(3, 282) = 3.181, *p* = .024, *η^2^p* = .033, the external perfectionism, *F*(3, 282) = 6.007, *p* < .001, *η^2^p* = .060, and the striving for excellence, *F*(3, 282) = 5.594, *p* < .001, *η^2^p* = .056, models were significant. A main effect of age emerged for amotivation, *F*(1, 282) = 6.354, *p* = .012, *η^2^p* = .023, and external perfectionism, *F*(1, 282) = 6.991, *p* = .009, *η^2^p* = .056, such that an increase in age was associated with a decrease in amotivation and in external perfectionism. For striving for excellence, the main effect of age, *F*(1, 282) = 16.378, *p* < .001, *η^2^p* = .037 was qualified by an interaction with gender, *F*(1, 282) = 5.793, *p* = .017, *η^2^p* = .020. Simple effect analyses revealed that men, *F*(1, 282) = 12.564, *p* < .001, *η^2^p* = .043, showed a greater increase in striving for excellence with age than women, *F*(1, 282) = 3.927, *p* = .048, *η^2^p* = .014. The models overall respected all assumptions (see Figures S7 to S15).

**Table S6**

GLM of Amotivation, External Perfectionism and Striving for Excellence by Gender and Age (Study 2)

|  | Estimate | SE | 95% IC | | | | *p* | | β |
| --- | --- | --- | --- | --- | --- | --- | --- | --- | --- |
|  |  |  | LL | | UL | |  | |  |
|  |  |  |  | |  | |  | |  |
| *Amotivation* | | | | | | | | | |
| Gender | -.334 | .229 | -.784 | .116 | | .145 | | -.227 | |
| Age | -.032 | .013 | -.058 | - .007 | | .012 | | -.196 | |
| Gender x Age | -.024 | .026 | -.074 | .027 | | .355 | | -.144 | |
|  | | | | | | | | | |
| *External Perfectionism* | | | | | | | | | |
| Gender | -.200 | .196 | -.586 | .186 | | .308 | | -.156 | |
| Age | -.029 | .011 | -.051 | - .007 | | .009 | | -.203 | |
| Gender x Age | .014 | .022 | -.029 | .057 | | .525 | | .097 | |
|  | | | | | | | | | |
| *Striving for Excellence* | | | | | | | | | |
| Gender | .094 | .199 | -.298 | .486 | | .638 | | .072 | |
| Age | .045 | .011 | .023 | .067 | | <.001 | | .311 | |
| Gender x Age | .054 | .022 | .010 | .098 | | .017 | | .370 | |
| Men | .072 | .020 | .032 | .112 | | <.001 | |  | |
| Women | .018 | .009 | 1.226e-4 | .036 | | .048 | |  | |

**Figure S7**

*
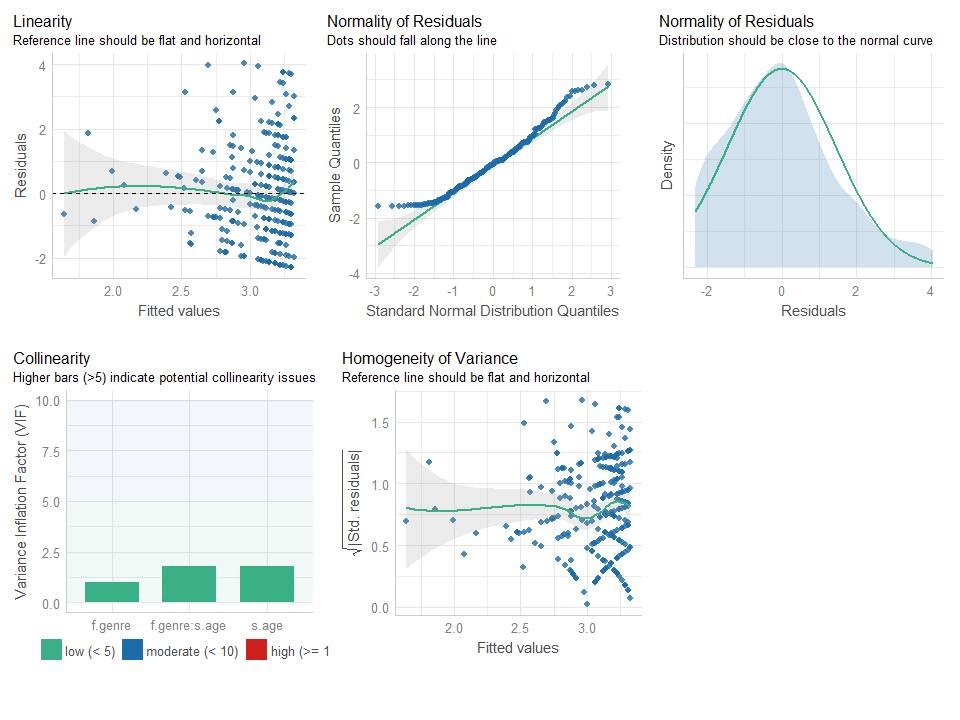
Collinearity and Homogeneity, Linearity and Normality of Residuals of Amotivation by Age and Gender for Study 2*

***Figure S8***

*Leverage and Cook’s Distance of Amotivation by Age and Gender for Study 2*

**
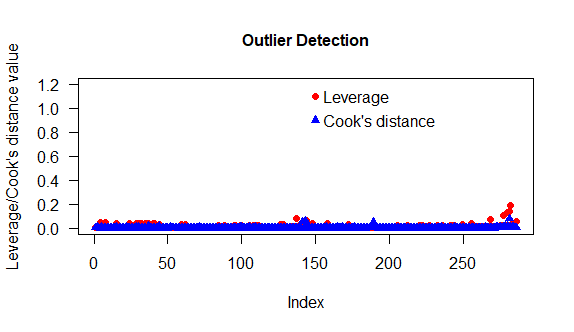
**

***Figure S9***

*Residuals per Predictor of Amotivation by Age and Gender for Study 2*

*
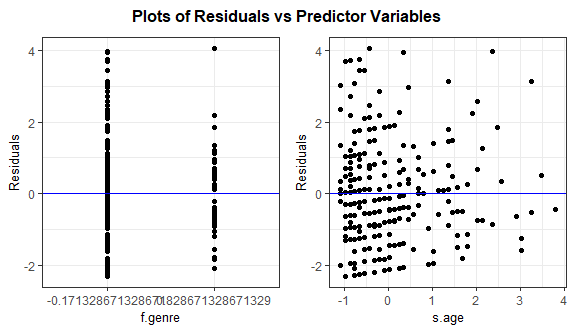
*

**Figure S10**

*
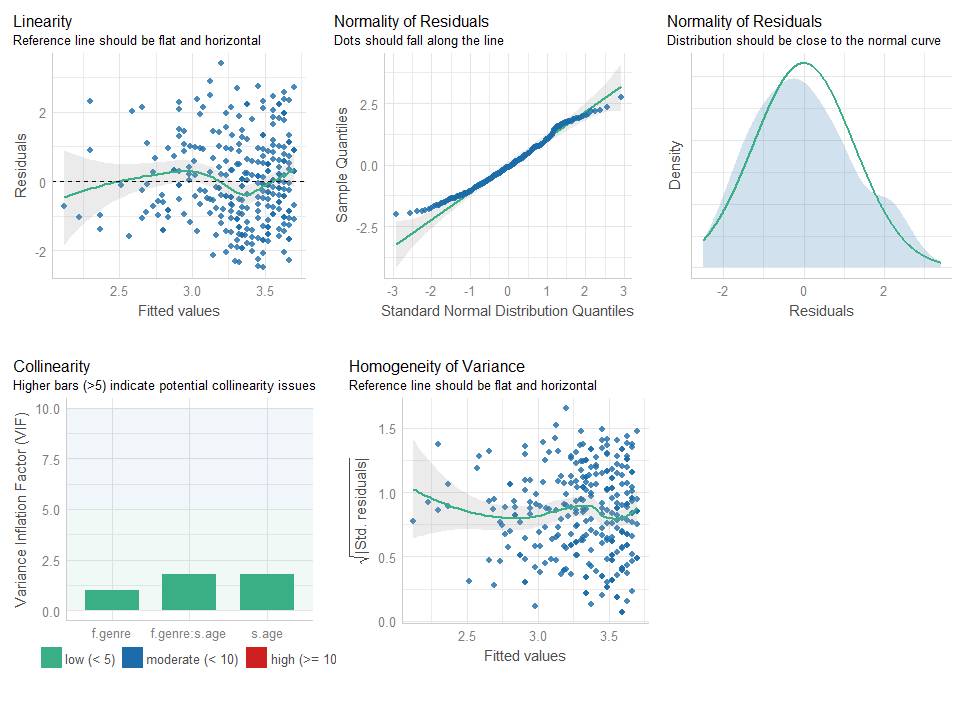
Collinearity and Homogeneity, Linearity and Normality of Residuals of External Perfectionism by Age and Gender for Study 2*

***Figure S11***

*Leverage and Cook’s Distance of External Perfectionism by Age and Gender for Study 2*

**
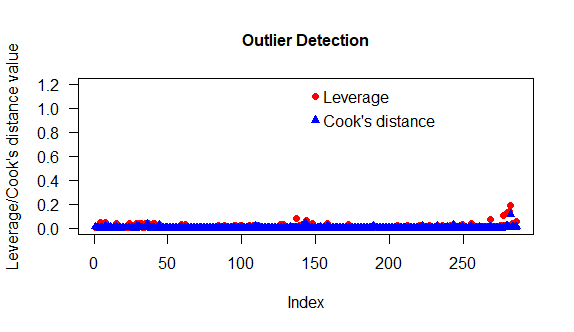
**

***Figure S12***

*Residuals per Predictor of External Perfectionism by Age and Gender for Study 2*

*
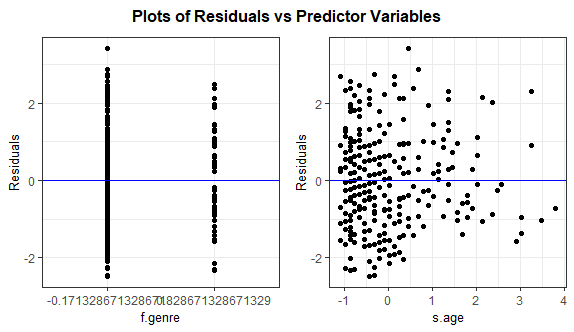
*

**Figure S13**

*
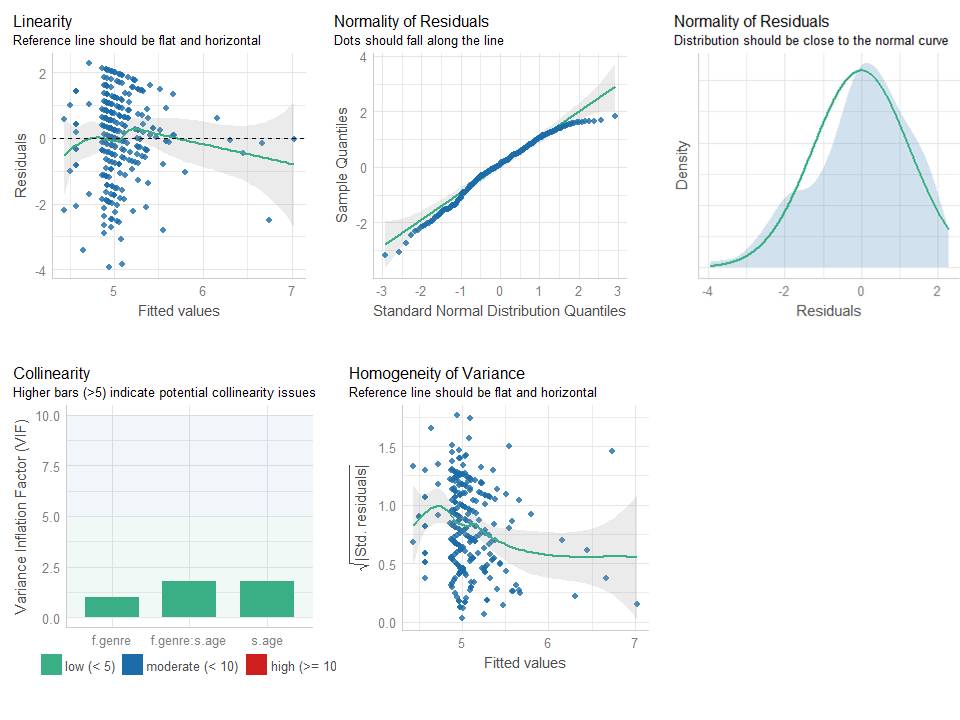
Collinearity and Homogeneity, Linearity and Normality of Residuals of Striving for Excellence by Age and Gender for Study 2*

***Figure S14***

*Leverage and Cook’s Distance of Striving for Excellence by Age and Gender for Study 2*

**
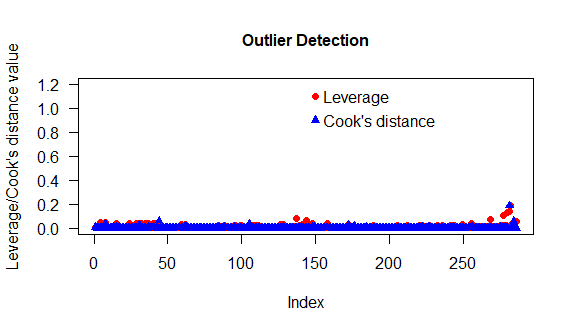
**

***Figure S15***

*Residuals per Predictor of Striving for Excellence by Age and Gender for Study 2*

**
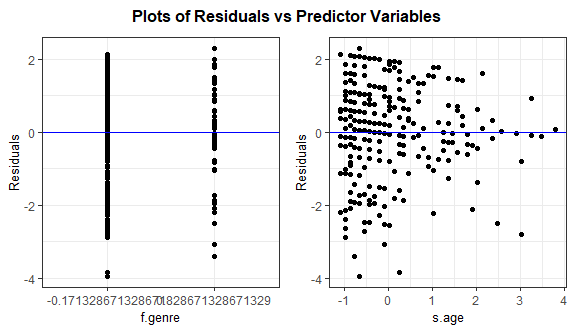
**

**References**

Goode, K., & Rey, K. (2019). *ggResidpanel: Panels and Interactive Versions of Diagnostic Plots using 'ggplot2'*. https://CRAN.R-project.org/package=ggResidpanel

Lüdecke, D., Ben-Shachar, M. S., Patil, I., Waggoner, P., & Makowski, D. (2021). performance: An R Package for Assessment, Comparison and Testing of Statistical Models. . *Journal of Open Source Software, 6*(60), 3139. https://doi.org/10.21105/joss.03139

R Core Team. (2021). *R: A language and environment for statistical computing.* R Foundation for Statistical Computing. https://www.R-project.org/

RStudio Team. (2020). *RStudio: Integrated Development Environment for R.* http://www.rstudio.com/

Silk, M. (2019). *Mixed Model Diagnostics*. https://dfzljdn9uc3pi.cloudfront.net/2020/9522/1/MixedModelDiagnostics.html

**
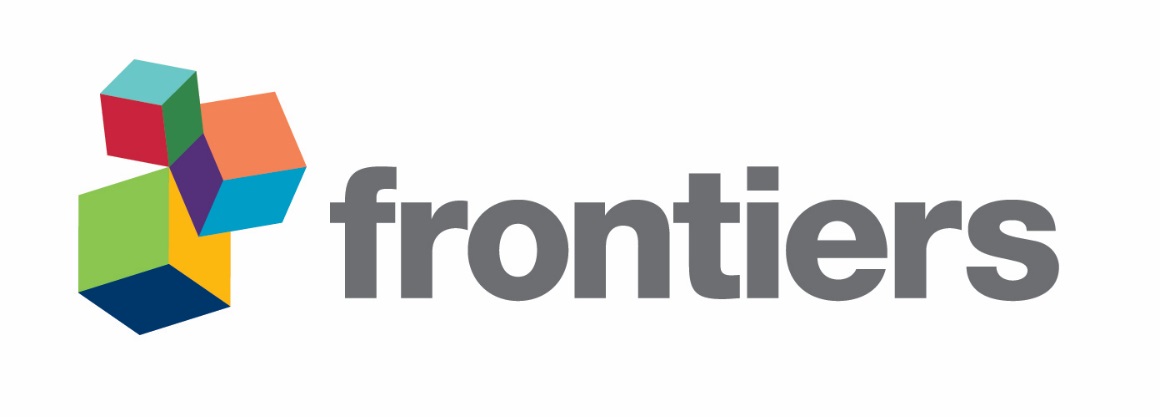
**
